# Small heat shock proteins HspB1 and HspB5 differentially alter the condensation and aggregation of the TDP‐43 low‐complexity domain

**DOI:** 10.1002/pro.70539

**Published:** 2026-03-19

**Authors:** Thomas B. Walker, Joshua W. Trowbridge, Shannon McMahon, Nicholas R. Marzano, Lauren Rice, Justin J. Yerbury, Heath Ecroyd, Luke McAlary

**Affiliations:** ^1^ Molecular Horizons and School of Science University of Wollongong Wollongong New South Wales Australia

**Keywords:** amyotrophic lateral sclerosis, chaperones, fibrillation, liquid–liquid phase separation, proteostasis

## Abstract

TAR DNA‐binding protein 43 (TDP‐43) is a nucleic acid‐binding protein that regulates processes of mRNA metabolism, during which it undergoes condensation mediated by its C‐terminal low‐complexity domain (TDP‐43^LCD^). TDP‐43 aggregation and condensation are associated with neurodegenerative disease. However, the proteostasis mechanisms that regulate these processes remain elusive. Some evidence has shown that the molecular chaperone small heat shock protein HspB1 binds to and regulates the cytoplasmic phase separation of TDP‐43, indicating that other small heat shock proteins may have similar effects. Here, we demonstrate divergent behaviors for HspB1 and its homolog HspB5 on TDP‐43^LCD^ condensation and aggregation. In addition to inhibiting TDP‐43^LCD^ aggregation, HspB1 partitions into TDP‐43^LCD^ condensates and increases the dynamic exchange of TDP‐43^LCD^ within condensates and with the surrounding solution. Phosphorylation‐mimicking mutations within HspB1 enhance these effects. HspB5 inhibits TDP‐43^LCD^ aggregation more effectively than HspB1 and partitions into TDP‐43^LCD^ condensates, where it delays the pathological transition of the condensate to a gel/solid. We identify the N‐ and C‐terminal regions of HspB1 and HspB5 to be crucial for the chaperone effects, and highlight the role of sequence diversity within these regions in defining small heat shock protein function. These findings demonstrate that HspB1 and HspB5 are regulators of TDP‐43 phase separation and aggregation and may be potential therapeutic targets in mitigating toxic TDP‐43 aggregation in neurodegenerative disease.

## 1 INTRODUCTION

Transactive response (TAR) DNA‐binding protein of 43 kDa (TDP‐43) is a nucleic acid‐binding protein primarily involved in RNA metabolism (Prasad et al., 2019). Predominantly localized within the nucleus, TDP‐43 mislocalizes to the cytoplasm of affected cells in amyotrophic lateral sclerosis (ALS) and frontotemporal dementia associated with TDP‐43 pathology (FTLD‐TDP), where it exists as a major component of pathological proteinaceous inclusions (Arai et al., 2006; Ling et al., 2013). Roughly 22 missense mutations in TDP‐43 have been curated as likely pathogenic or pathogenic for ALS and/or FTLD‐TDP. Most of these variants are situated within the TDP‐43 low‐complexity domain (TDP‐43^LCD^), a region that governs liquid–liquid phase separation and is aggregation‐prone (McAlary et al., 2019; Tamaki & Urushitani, 2022), highlighting its potential role in the progression of TDP‐43 proteinopathies. Experimental evidence indicates that biomolecular condensates formed via phase separation of TDP‐43 may be involved in the pathological mechanisms of aggregation, in which TDP‐43 is believed to undergo a phase transition from a dynamic, liquid‐like state toward more static, gel‐like states (Babinchak et al., 2019; Haider et al., 2024; Mann et al., 2019; Mohanty et al., 2023; Mohanty, Shenoy, Rizuan, Mercado‐Ortiz, et al., 2023; Pakravan et al., 2021; Staderini et al., 2022; Staderini et al., 2024). The mechanisms underlying this process are distinct from classical amyloid fibrillation, in which misfolded proteins rich in β‐sheet structures self‐assemble into pre‐fibrillar oligomers that elongate by irreversibly binding monomers, eventuating in long insoluble fibrils (Rambaran & Serpell, 2008). Therefore, to illuminate potential therapeutic strategies against aggregation in ALS and FTLD, it is important to understand the molecular mechanisms that regulate condensation and aggregation of TDP‐43.

A core element of protein homeostasis (proteostasis) is molecular chaperones, which are a diverse family of proteins that mitigate protein misfolding. Small heat shock proteins (sHsps) are an important class of molecular chaperones that bind to misfolded proteins and sequester them into large soluble complexes in an ATP‐independent manner (Basha et al., 2012; Nakamoto & Vigh, 2007). sHsps are characterized by a central β‐sheet‐rich α‐crystallin domain (ACD), which is flanked by disordered N‐ and C‐terminal regions important for the recognition of client proteins and assembly into large polydisperse oligomers (Carver et al., 1992; Janowska et al., 2019; Kampinga et al., 2009; Kim et al., 1998; Van Montfort et al., 2001). Of the 10 sHsps, HspB1 (also known as Hsp27), and HspB5 (also known as αB‐crystallin) are the most widely studied, in part because they have been found to colocalize with astrocytic inclusions in patients with familial ALS (Kato et al., 1997) and due to their widespread expression and robust chaperone function. These sHsps natively exist as large polydisperse hetero‐oligomers of up to 40 subunits (Arrigo & Gibert, 2013; Basha et al., 2012; Boelens, 2020). Phosphorylation of serine residues within the N‐terminal domains of HspB1 and HspB5 causes the dissociation of larger oligomers into smaller, more chaperone‐active forms, thus enhancing their function during periods of cellular stress (Ahmad et al., 2008; Aquilina et al., 2004; Aquilina et al., 2013; Ecroyd et al., 2007; Ito et al., 2001; Jovcevski et al., 2015; Peschek et al., 2013). In line with this, both HspB1 and HspB5 have been found to attenuate aggregation of multiple protein substrates in vitro and *in cyto*, a mechanism enhanced by sHsp variants that mimic physiological phosphorylation (Cox et al., 2016; Cox & Ecroyd, 2017; Hochberg et al., 2014; Jovcevski et al., 2015; Selig et al., 2020).

Despite their importance to proteostasis, the investigation of HspB1 and HspB5 as regulators of TDP‐43 aggregation has been minimal. Indeed, it was only recently demonstrated that HspB1 regulates the condensation of TDP‐43 within the cytoplasm (Lu et al., 2022; Yamashita et al., 2025). To our knowledge, no such investigations have been undertaken for HspB5, or work to address the impact of these sHsps on the aggregation of TDP‐43 in the absence of condensation. To address these gaps, we examined the roles of HspB1 and HspB5 in regulating the fibrillation and condensation of a purified recombinant form of TDP‐43^LCD^, comprising amino acids 267–414 of TDP‐43. We found through kinetic aggregation assays that HspB5 is substantially more effective than HspB1 at preventing the fibrillar aggregation of TDP‐43^LCD^ and that the ACD of HspB5 is involved in suppressing fibrillation. Furthermore, phosphomimetic mutant forms of HspB1 and HspB5 also inhibit TDP‐43^LCD^ fibrillation. Additionally, we found that HspB1 promotes the condensation of the TDP‐43^LCD^ and that this behavior is enhanced for phosphomimetic HspB1. Using FRAP, we showed that phosphomimetic HspB1 enhances TDP‐43^LCD^ condensate dynamics but does not prevent condensates from undergoing pathological phase transitions. On the other hand, phosphomimetic mutant HspB5 does not promote TDP‐43^LCD^ condensation; rather, it enhanced condensate dynamics and inhibited condensate phase transitions. Overall, the results indicate that HspB1 and HspB5 both act to prevent TDP‐43^LCD^ phase transition and fibrillation; however, they do so to different extents.

## 2 RESULTS

### 2.1 The TDP‐43 low complexity domain (TDP‐43^LCD^ ) forms amyloid fibrils in kinetic aggregation assays

**FIGURE 1:**
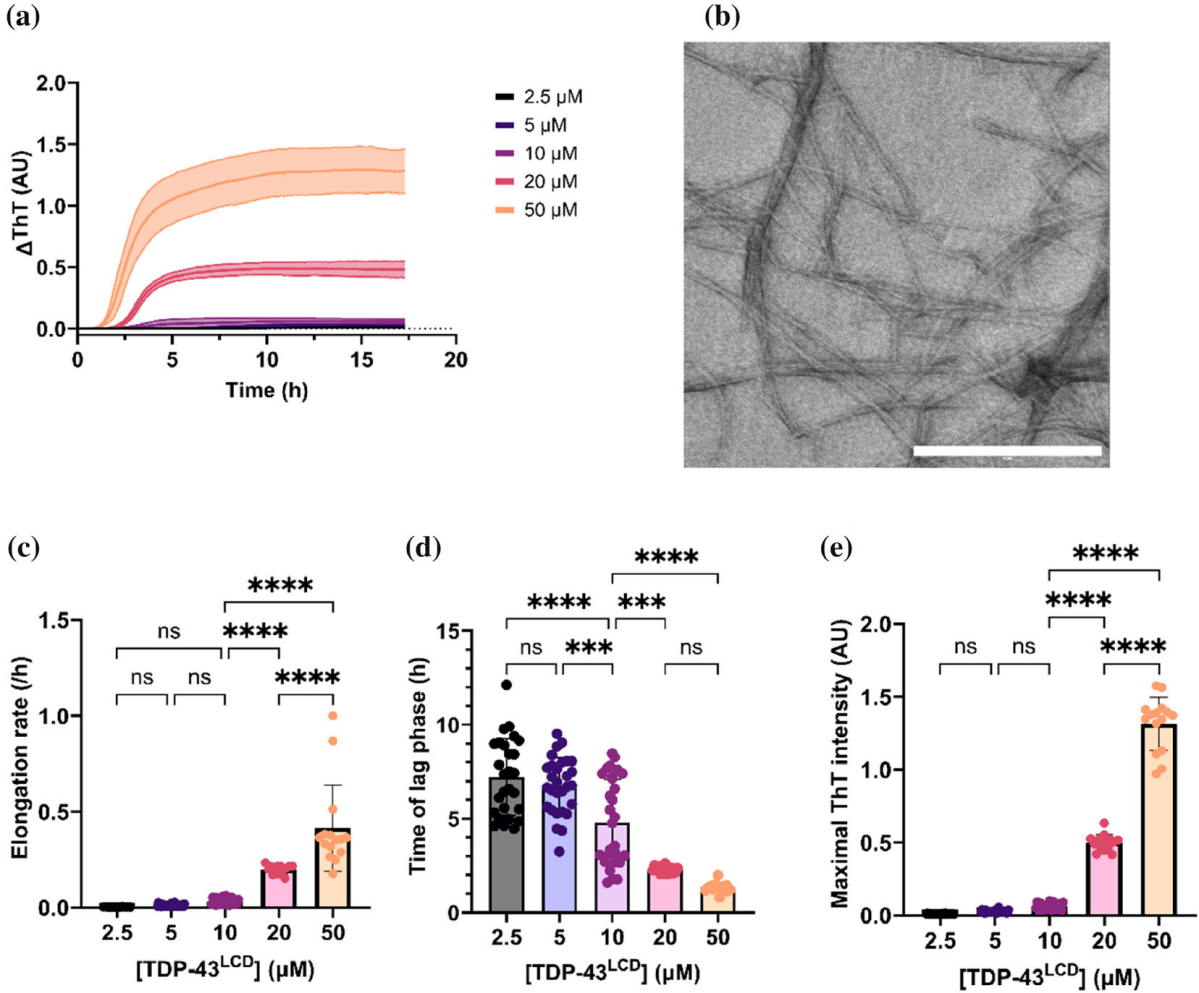
Fibrillation of TDP‐43^LCD^ is concentration‐dependent. Increasing concentrations of TDP‐43^LCD^ (0–50 μM) were incubated with 25 μM ThT at 37°C and changes in ThT fluorescence at 490 nm were measured over time. Samples were shaken at 200 rpm for 30 s prior to each cycle. (a) ThT curves for different concentrations of TDP‐43^LCD^ (gain = 1500). (b) Negative stain electron microscope (EM) images of TDP‐43^LCD^ fibrils. Scale bar represents 500 nm. (c–e) ThT intensity curves were used to calculate (c) elongation rate; (d) the time of lag phase; and (e) maximal ThT intensity. Data are plotted as mean ± SD (*n* ≥ 15) and analyzed by one‐way ANOVA with Tukey's post‐hoc test (*p < 0.05, **p < 0.01, ***p < 0.001, ****p < 0.0001).

Since the C‐terminal LCD of TDP‐43 harbors an amyloidogenic core region that is essential for promoting fibrillation (Conicella et al., 2020; Jiang et al., 2013; Johnson et al., 2009), we first sought to determine the propensity of TDP‐43^LCD^ to aggregate using an agitated in vitro thioflavin‐T (ThT) assay. We performed these assays in the absence of salt and at a pH slightly lower than physiological (pH 6.0) as these conditions were previously established to allow appreciable concentrations of TDP‐43^LCD^ to be present without undergoing spontaneous phase‐transition (Conicella et al., 2016; Conicella et al., 2020; Li, Chen, et al., 2018; Li, Chiang, et al., 2018). We observed an increase in ThT fluorescence over roughly 16 h with increasing concentrations of TDP‐43^LCD^ (Figure 1a). Electron microscopy of samples at the end of this assay revealed the increase in ThT fluorescence was due to the formation of fibrils (Figure 1b). Since there appeared to be concentration‐dependent differences in the extent of fibril formation, ThT data were fitted to a Boltzmann sigmoidal curve to derive their elongation rate (i.e., the rate of fibril growth) and time of lag phase (i.e., the time until an increase in fluorescence is observed). Elongation rate was greater with increasing concentrations of TDP‐43^LCD^ (Figure 1c). We observed that solutions containing the highest concentrations of TDP‐43^LCD^ quickly (within minutes of incubation) became turbid (Figure S1), indicating that condensation had likely taken place as described in previous work (Conicella et al., 2016; Conicella et al., 2020), thus preventing some monomeric TDP‐43^LCD^ from forming fibrils. Despite this, we observed a decrease in the time of lag phase as the concentration of TDP‐43^LCD^ increased (Figure 1c). Additionally, we observed a concentration‐dependent increase in maximal ThT fluorescence intensity at end point (Figure 1d). These data are consistent with TDP‐43^LCD^ undergoing a nucleation‐dependent process of fibril formation under these incubation conditions, accordant with the general model by which amyloid fibrils form (Johnson et al., 2009). Having noted the effect of protein concentration and condensation upon the kinetics of TDP‐43^LCD^ fibrillation, we performed all subsequent aggregation assays using 10 μM TDP‐43^LCD^ to mitigate any confounding effects from its phase separation.

### 2.2 HspB5 inhibits TDP‐43^LCD^ fibrillation by prolonging the lag phase and slowing elongation to a greater extent than HspB1

**FIGURE 2:**
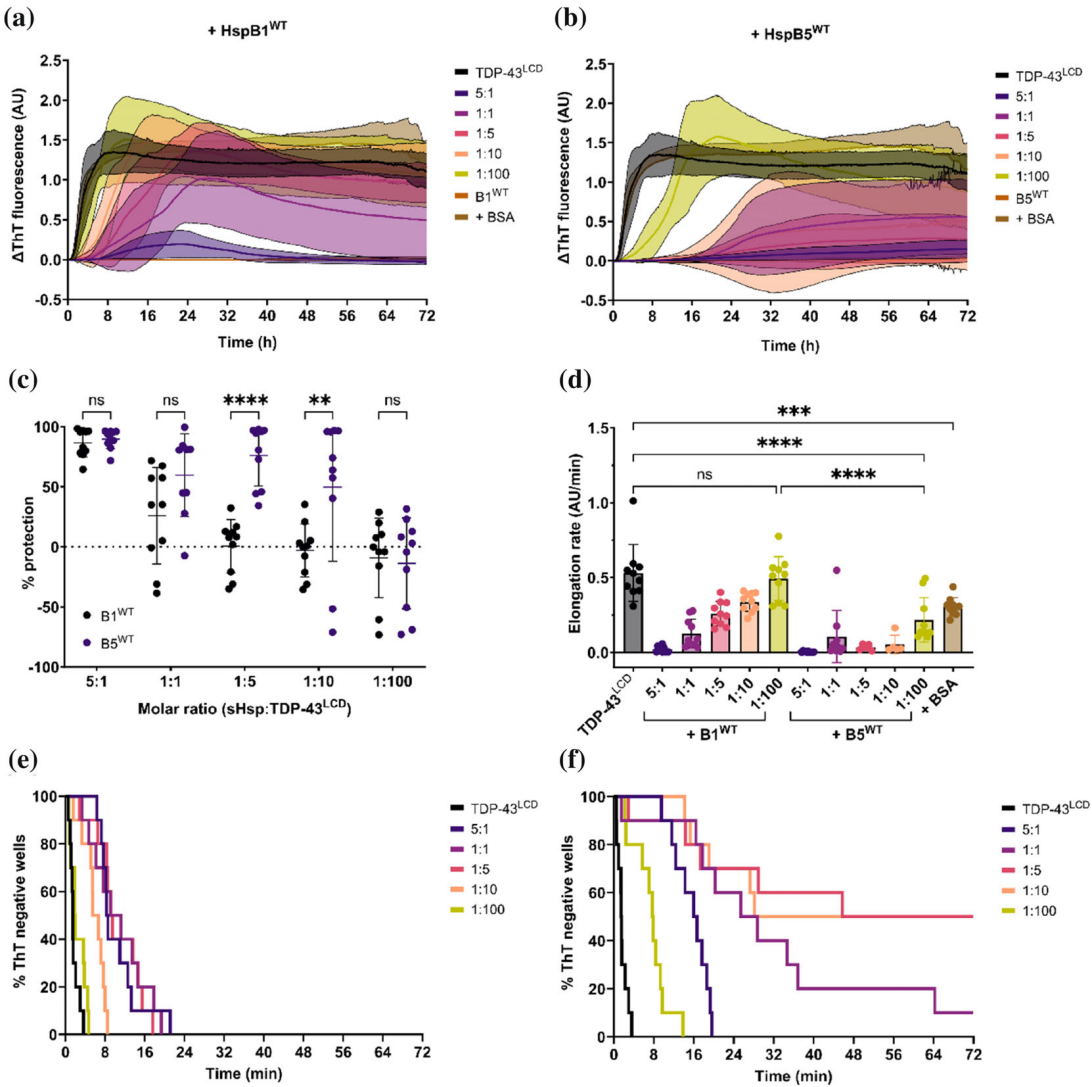
HspB5^WT^ is a more effective inhibitor of TDP‐43^LCD^ fibrillation than HspB1^WT^. TDP‐43^LCD^ (10 μM) was incubated with HspB1^WT^ or HspB5^WT^ at different molar ratios (5:1, 1:1, 1:5, 1:10, 1:100; sHsp:TDP‐43^LCD^). Samples were incubated in the presence of 25 μM ThT at 37°C and monitored for changes in ThT fluorescence at 490 nm. Samples were shaken at 200 rpm for 30 s prior to each cycle. (a,b) ThT curve for TDP‐43^LCD^ in the presence of (a) HspB1^WT^ and (b) HspB5^WT^. Gain = 1700. (c) Percentage protection afforded by HspB1^WT^ and HspB5^WT^ against TDP‐43^LCD^ fibrillation. Protection was calculated relative to the mean maximal ThT fluorescence intensity of TDP‐43^LCD^ alone. (d) Elongation rate of TDP‐43^LCD^ fibrils in the presence of HspB1^WT^, HspB5^WT^, or BSA. (e) Kaplan–Meier analysis of TDP‐43^LCD^ fibrillation in the presence of HspB1^WT^. (f) Kaplan–Meier analysis of TDP‐43^LCD^ fibrillation in the presence of HspB5^WT^. Data plotted as mean ± SEM (a, b; *n* ≥ 9 technical replicates from two independent experiments) or mean ± SD (c,d; *n* ≥ 3 technical replicates from two independent experiments). Data analyzed by (c) two‐way ANOVA with Šidak's post‐hoc test or (d) one‐way ANOVA with Tukey's post‐hoc test (**p < 0.01, ***p < 0.001, ****p < 0.0001).

HspB1 has been shown to directly bind the LCD of TDP‐43 and prevent its assembly into aggregates in cells (Lu et al., 2022). However, it remains to be established whether HspB1 or HspB5 can alter the kinetics of TDP‐43 aggregation in assays using purified recombinant proteins. Therefore, we performed a kinetic ThT assay evaluating the aggregation of TDP‐43^LCD^ in the presence of varying concentrations of wild‐type forms of HspB1, HspB5, or, as a negative control, bovine serum albumin (BSA). As expected, incubation of TDP‐43^LCD^ alone resulted in an increase in ThT fluorescence over roughly 72 h in a sigmoidal manner, indicative of the formation of TDP‐43^LCD^ fibrils (Figure 2). Incubation of TDP‐43^LCD^ with non‐chaperone control BSA did not result in any significant change in ThT fluorescence compared to TDP‐43^LCD^ alone (Figure 2a,b). However, incubation of TDP‐43^LCD^ with HspB1^WT^ or HspB5^WT^ at molar ratios ranging from 5:1 to 1:100 (sHsp:TDP‐43^LCD^) inhibited the increase in ThT fluorescence in a concentration‐dependent manner (Figure 2a,b). There was no increase in ThT fluorescence when either of the sHsps were incubated in the absence of TDP‐43^LCD^.

The percentage protection afforded against TDP‐43^LCD^ fibrillation by HspB1^WT^ and HspB5^WT^ was similar at molar ratios of 5:1 or 1:1 (sHsp:TDP‐43^LCD^) but was greater for HspB5^WT^ at 1:5 and 1:10 molar ratios, suggesting that HspB5^WT^ more potently inhibits the fibrillation of TDP‐43^LCD^ (Figure 2c). At lower molar ratios, the presence of the sHsps sometimes resulted in higher levels of ThT fluorescence compared to TDP‐43^LCD^ alone (measured as negative percent protection in Figure 2c), indicative of the inability of the chaperones to prevent the overall amount of TDP‐43^LCD^ fibril formation at these molar ratios. Fitting to a Boltzmann sigmoidal curve revealed that the presence of the sHsp also led to a decrease in the elongation rate of TDP‐43^LCD^ fibrils, indicating that both HspB1^WT^ and HspB5^WT^ impede the growth of TDP‐43^LCD^ fibrils (Figure 2d). Even at molar ratios as low as 1:100, HspB5^WT^ was capable of significantly reducing the elongation rate of TDP‐43^LCD^ fibrillation compared to TDP‐43^LCD^ alone, while HspB1^WT^ had no effect on the elongation rate at this same molar ratio. While BSA significantly reduced the elongation rate of TDP‐43^LCD^ fibrils, this only occurred at the relatively high molar ratio of 1:1. Thus, these data indicate that HspB5^WT^ is more effective than HspB1^WT^ at reducing TDP‐43^LCD^ fibril formation under these conditions.

A Kaplan–Meier analysis of the lag‐phase of aggregation was performed as described previously (Abdolvahabi et al., 2017) to assess the impact of the chaperones on the initiation of fibril formation by TDP‐43^LCD^ within individual wells of the assay. In the presence of HspB1^WT^, the onset of TDP‐43^LCD^ fibrillation was significantly delayed at molar ratios as low as 1:10 (sHsp:TDP‐43^LCD^), as indicated by the right‐shift in the Kaplan–Meier distributions (Figure 2e). The onset of TDP‐43^LCD^ fibrillation was significantly delayed in the presence of HspB5^WT^ at each of the molar ratios tested (Figure 2f). Indeed, HspB5^WT^ consistently conferred a lower hazard ratio than HspB1^WT^ at each molar ratio tested, indicating that it more effectively reduced the likelihood of TDP‐43^LCD^ fibrillation (Table S2). Despite not observing an increase in ThT signal across our assay time, we do note that fibrillation could simply take longer under our assay conditions and not be fully suppressed. Altogether, these data indicate that HspB5^WT^ is a potent inhibitor of the formation and growth of TDP‐43^LCD^ fibrils.

### 2.3 Phosphomimetic isoforms of HspB1 (HspB1^3D^ ) and HspB5 (HspB5^3D^ ) inhibit TDP‐43^LCD^ fibrillation

**FIGURE 3:**
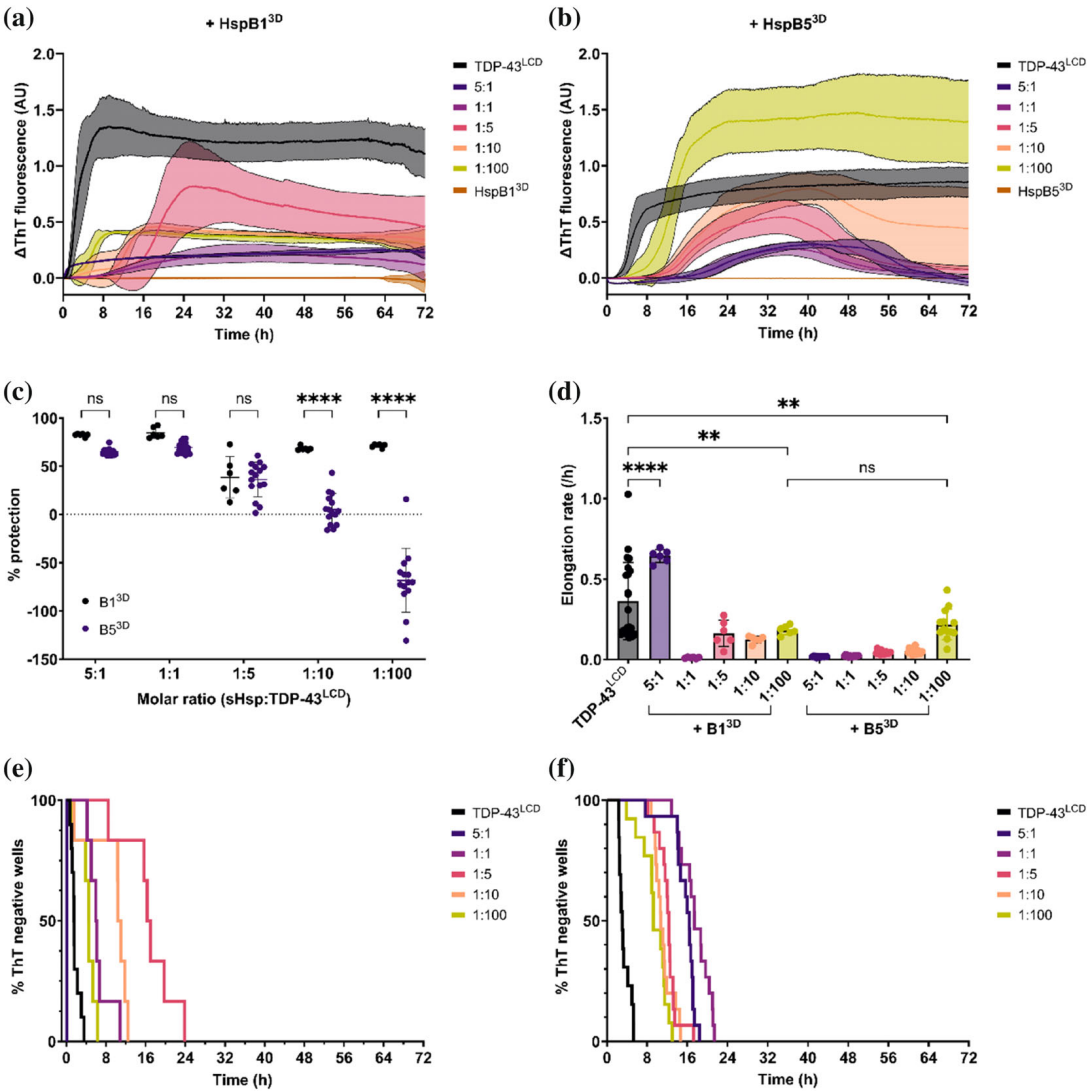
The effect of phosphomimetic HspB1 and HspB5 on the fibrillation of TDP‐43^LCD^. TDP‐43^LCD^ (10 μM) was incubated with different molar ratios (5:1, 1:1, 1:5, 1:10, 1:100) of HspB1^3D^ or HspB5^3D^ (sHsp:TDP‐43^LCD^) and 25 μM ThT at 37°C and monitored for changes in ThT fluorescence at 490 nm. Samples were shaken at 200 rpm for 30 s prior to each cycle. (a) ThT curve for TDP‐43^LCD^ in the presence of HspB1^3D^. Assay was performed concurrently to assays shown in Figure 2. Gain = 1700. (b) Percentage protection against TDP‐43^LCD^ for HspB1^3D^ and HspB5^3D^.(d) Elongation rate of TDP‐43^LCD^ fibrils in the presence of HspB1^3D^ or HspB5^3D^. (e,f) Kaplan–Meier analysis of TDP‐43^LCD^ fibrillation in the presence of (e) HspB1^3D^ or (f) HspB5^3D^. Data plotted as mean ± SD (a–d) (*n* ≥ 5 technical replicates from two independent experiments). Data analyzed by (c) two‐way ANOVA with Šidak's post‐hoc test or (d) one‐way ANOVA with Tukey's post‐hoc test (**p < 0.01, ****p < 0.0001).

Previous studies have shown that phosphomimetic mutations at serines 15, 78, and 82 reduce the size of HspB1 homo‐oligomers, while similar mutations in HspB5 at serines 19, 45, and 59 also decrease oligomer size but to a lesser extent (Aquilina et al., 2004; Benesch et al., 2008; Ecroyd et al., 2007; Jovcevski et al., 2015). Furthermore, phosphomimetic HspB1 has been found to have enhanced chaperone activity against several aggregating proteins through increased exposure of substrate binding regions (Hayes et al., 2009; Jovcevski et al., 2015). We therefore sought to examine whether phosphomimetic forms of HspB1 and HspB5 (called HspB1^3D^ and HspB5^3D^, respectively) also inhibit the formation of TDP‐43^LCD^ fibrils. Incubation of TDP‐43^LCD^ with increasing amounts of HspB1^3D^ led to a decrease in ThT fluorescence intensity compared to when TDP‐43^LCD^ was incubated alone (Figure 3a). Similarly, incubation with HspB5^3D^ led to a change in ThT fluorescence intensity (Figure 3b). HspB1^3D^ protected against TDP‐43^LCD^ fibrillation at all molar ratios tested, consistent with it having greater chaperone activity than HspB1^WT^ (Ito et al., 2001). While higher molar ratios of HspB5^3D^ conferred similar protection against TDP‐43^LCD^ fibrillation as HspB1^3D^, it was ineffective at molar ratios of 1:10 and 1:100 (Figure 3c).

While HspB1^3D^ enhanced the elongation rate of TDP‐43^LCD^ fibrils at a 5:1 molar ratio, at lower concentrations it significantly reduced fibril growth (Figure 3d). In contrast, HspB5^3D^ reduced the elongation rate of TDP‐43^LCD^ fibrils at each of the molar ratios tested (Figure 3d). HspB1^3D^ delayed TDP‐43^LCD^ aggregation at all molar ratios except 5:1 (Figure 3e), while HspB5^3D^ delayed aggregation at all molar ratios tested (Figure 3f). Furthermore, HspB5^3D^ reduced the hazard ratio for TDP‐43^LCD^ aggregation to a greater extent than HspB1^3D^, suggesting that it was more effective in reducing the likelihood of aggregation (Table S2). These findings are consistent with previous work demonstrating that phosphomimetic mutant HspB1 exhibits greater chaperone activity than its wild‐type counterpart (Cox et al., 2016; Jovcevski et al., 2015) and demonstrate a similar effect for phosphomimetic mutant HspB5.

### 2.4 The α‐crystallin domain (ACDs) of HspB1 and HspB5 partially inhibit TDP‐43^LCD^ aggregation

**FIGURE 4:**
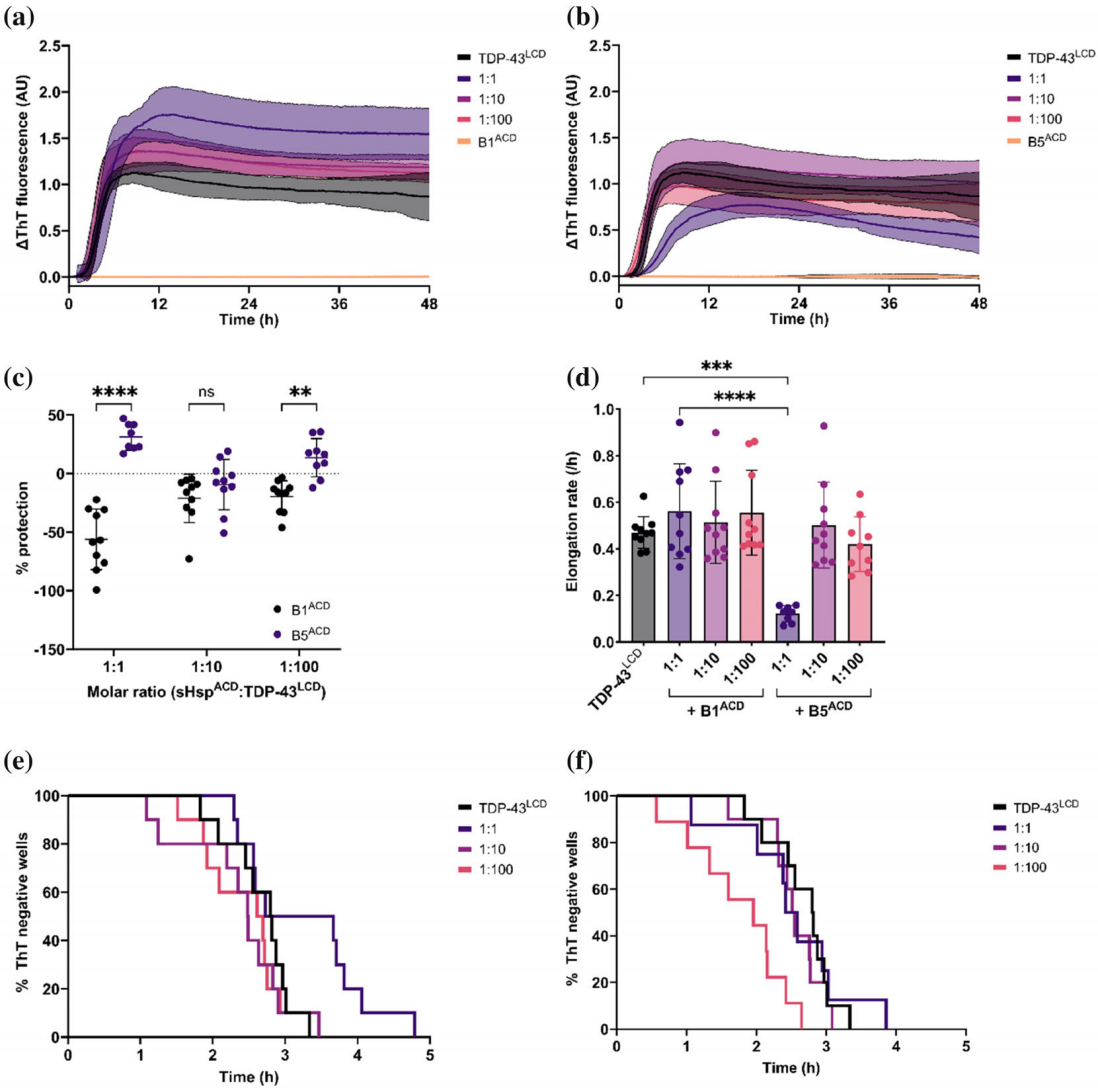
HspB1 and HspB5 partially inhibit TDP‐43^LCD^ fibrillation through their α‐crystallin domains. TDP‐43^LCD^ (10 μM) was incubated with different molar ratios (1:1, 1:10, 1:100) of HspB1^ACD^ or HspB5^ACD^ (sHsp:TDP‐43^LCD^) and 25 μM ThT at 37°C and monitored for changes in ThT fluorescence at 490 nm. Samples were shaken at 200 rpm for 30 s prior to each cycle. (a) ThT curve for TDP‐43^LCD^ in the presence of HspB1^ACD^. (b) ThT curve for TDP‐43^LCD^ in the presence of HspB5^ACD^. (c) Percentage protection against TDP‐43^LCD^ fibrillation for HspB1^ACD^ and HspB5^ACD^. Protection was calculated relative to the mean ThT fluorescence intensity of TDP‐43^LCD^ alone. (d) Elongation rate of TDP‐43^LCD^ fibrils in the presence of HspB1^ACD^ or HspB5^ACD^. (e) Kaplan–Meier analysis of TDP‐43^LCD^ fibrillation in the presence of HspB1^ACD^. (f) Kaplan–Meier analysis of TDP‐43^LCD^ fibrillation in the presence of HspB5^ACD^. Data plotted as mean ± SEM (a,b) or mean ± SD (c, d) (*n* = 10 technical replicates from two independent experiments. Data analyzed by (c) two‐way ANOVA with Šidak's post‐hoc test or (d) one‐way ANOVA with Tukey's post‐hoc test (**p < 0.01, ***p < 0.001, ****p < 0.0001).

Given that both the full‐length isoforms of the sHsps inhibit the aggregation of TDP‐43^LCD^, we next determined whether this is mediated by the conserved alpha‐crystallin domains (ACDs) of these sHsps. Surprisingly, at a 1:1 molar ratio (sHsp^ACD^:TDP‐43^LCD^) HspB1^ACD^ increased the maximum ThT fluorescence compared to when TDP‐43^LCD^ was incubated alone (Figure 4a). Importantly, control wells containing HspB1^ACD^ alone showed no increase in ThT signal, indicating that this increase in ThT fluorescence was associated with TDP‐43^LCD^ aggregation (Figure 4a). In contrast, levels of ThT fluorescence associated with TDP‐43^LCD^ fibril formation were reduced in the presence of HspB5^ACD^ at a 1:1 molar ratio (sHsp^ACD^:TDP‐43^LCD^) (Figure 4b). Thus, in these experiments, HspB5^ACD^ conferred greater protection against TDP‐43^LCD^ aggregation than HspB1^ACD^; although it provided only modest protection at a 1:1 molar ratio and had little to no effect at 1:10 and 1:100 molar ratios (Figure 4c).

Additionally, while HspB1^ACD^ had no effect upon the elongation rate of TDP‐43^LCD^ fibrils at any of the molar ratios tested, HspB5^ACD^ reduced the elongation rate at a 1:1 molar ratio (Figure 4d). A Kaplan–Meier analysis demonstrated that TDP‐43^LCD^ aggregation was less likely to occur in the presence of an equimolar amount of HspB1^ACD^, which yielded a significantly reduced hazard ratio at this molar ratio; however, no difference was observed at a 1:10 or 1:100 ratio (HspB1^ACD^:TDP‐43^LCD^) (Figure 4e, Table S2). In contrast, TDP‐43^LCD^ aggregation was relatively unaffected by HspB5^ACD^ at a 1:1 or 1:10 molar ratio but was accelerated at a 1:100 molar ratio (Figure 4f). Under these conditions, HspB5^ACD^ conferred a much greater hazard ratio, indicating that it was increasing the likelihood of TDP‐43^LCD^ aggregation (Table S2). These findings suggest that the ACDs of these two sHsps play only a relatively minor role in the inhibition of the aggregation of TDP‐43^LCD^ into fibrils, in contrast to their disordered terminal regions. Instead, the ACDs of HspB1 and HspB5 appear to be involved in binding of these chaperones to TDP‐43^LCD^ (Figures S2 and S3).

### 2.5 Sodium chloride induces the condensation of the TDP‐43^LCD^ in a concentration‐dependent manner

**FIGURE 5:**
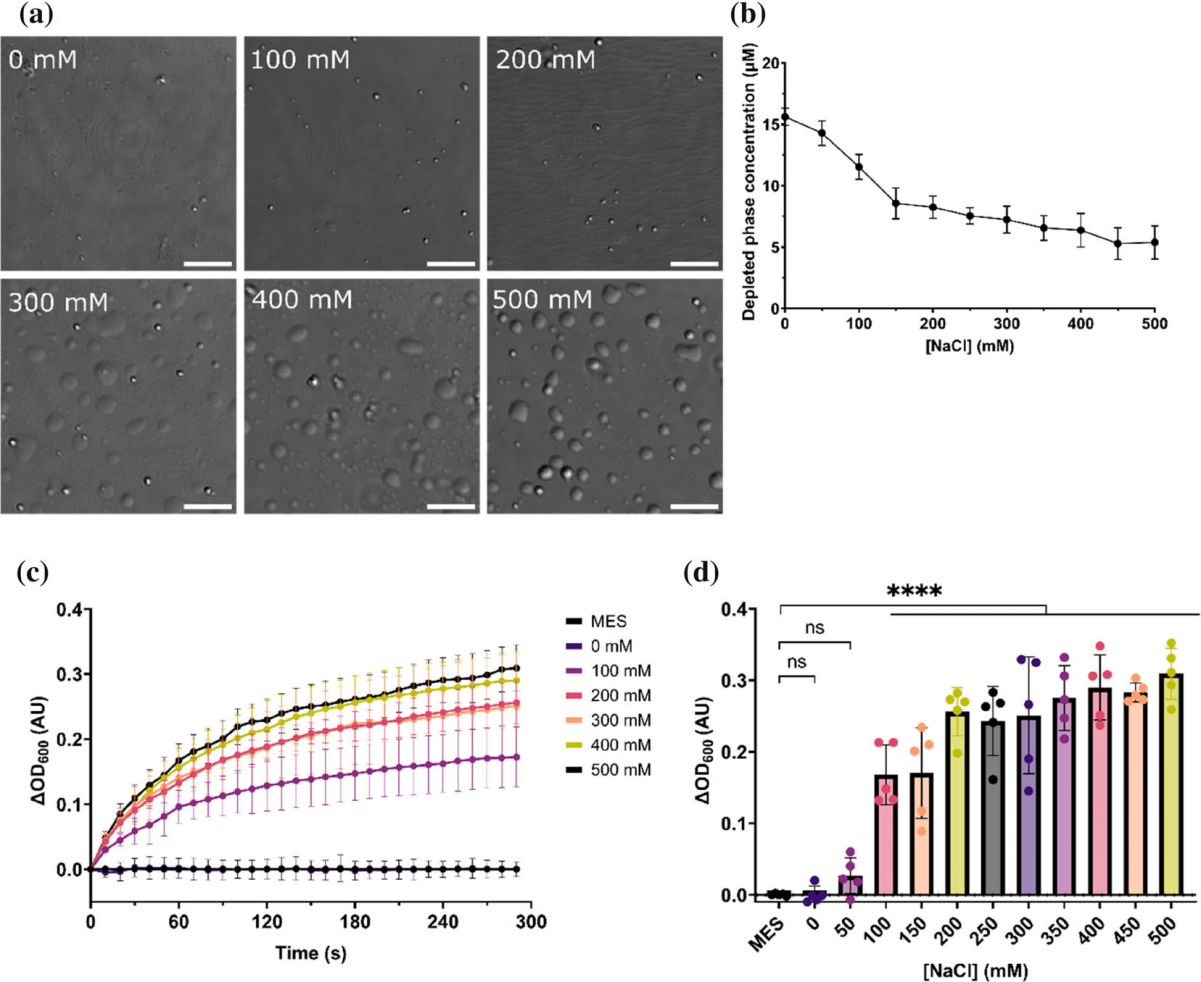
TDP‐43^LCD^ phase separates in a NaCl concentration‐dependent manner. TDP‐43^LCD^ (20 μM) in 20 mM MES (pH 6.0) was incubated with different concentrations of NaCl (0–500 mM) to induce phase separation. (a) Differential interference contrast (DIC) microscopy images of TDP‐43^LCD^ condensates. NaCl concentrations are indicated. Scale bars represent 10 μm. (b) Quantification of proportion of TDP‐43^LCD^ that remains in the dilute phase (supernatant) following incubation. (c,d) Quantification of the change in OD at 600 nm following mixing of TDP‐43^LCD^ with NaCl. (c) Change in OD as a function of time with increasing NaCl concentration. (d) Endpoint OD values with increasing NaCl concentration. Data plotted as mean ± SD (*n* = 5 technical replicates from 2 independent experiments). Data analyzed by one‐way ANOVA with Dunnett's post‐hoc test with comparison to MES control (**p < 0.01, ****p < 0.0001).

In addition to its role in the pathological aggregation of TDP‐43 in ALS and FTLD, TDP‐43^LCD^ has been shown to be sufficient and necessary for TDP‐43 condensation (Conicella et al., 2020). Condensation of TDP‐43^LCD^ is promoted by multiple factors including increased salt concentration, decreased temperature, pH, molecular crowding, and the presence of RNA (Conicella et al., 2016; Conicella et al., 2020; Jiang et al., 2013; Li, Chen, et al., 2018; Li, Chiang, et al., 2018; Lu et al., 2022). We sought to characterize the salt‐induced phase separation behavior of TDP‐43^LCD^ in our system. Purified TDP‐43^LCD^ (20 μM) was incubated in the presence of increasing concentrations of NaCl (0–500 mM) for 1 h at room temperature before an aliquot was loaded onto a glass slide for confocal imaging. In the absence of NaCl, small and sparsely abundant droplets were observed on the glass surface (Figure 5a); these were observed to become much larger and more abundant with increasing concentrations of NaCl. These droplets exhibited liquid‐like behavior—they were found to fuse with one another and wet the surface of the glass coverslip. Quantification of the protein concentration in the dilute phase (i.e., non‐condensate fraction) of the condensation reactions revealed a decrease in the concentration of TDP‐43^LCD^ with increasing NaCl concentrations, indicating increased incorporation of TDP‐43^LCD^ into condensates (Figure 5b). A kinetic light scattering assay was performed in which increasing optical density (OD) at 340 nm, 400 nm and 600 nm was used as an indication of the formation of condensates of increasing size. As expected, increasing salt concentration resulted in more rapid formation of condensates of various sizes, as the rate of condensate formation appeared lower for larger condensates (Figure 5c–e).

### 2.6 HspB1^3D^ promotes TDP‐43^LCD^ condensation

**FIGURE 6:**
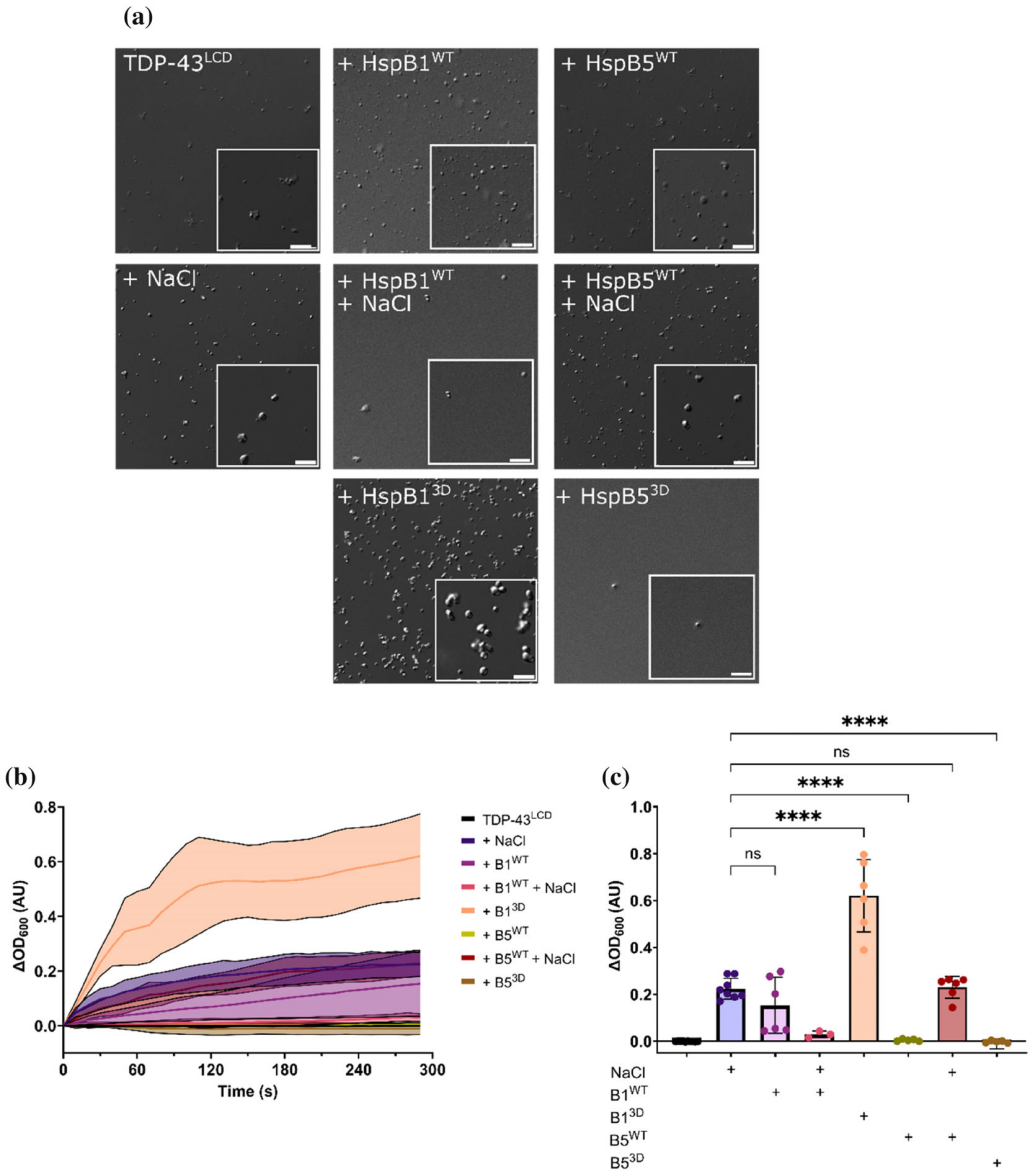
HspB1 promotes TDP‐43^LCD^ phase separation. TDP‐43^LCD^ (20 μM) in 20 mM MES (pH 6.0) was incubated in the presence of HspB1^3D^ (20 μM) or HspB5^WT^ (20 μM) in the presence or absence of NaCl (200 mM) (a) Differential interference contrast (DIC) confocal microscopy images of TDP‐43^LCD^ condensates formed in the presence of sHsps. Scale bars represent 5 μm. (b,c) Quantification of the change in OD at nm following mixing of TDP‐43^LCD^ with sHsps. (b) Change in OD for TDP‐43^LCD^ as a function of time in the presence of sHsps (c) Endpoint OD values for TDP‐43^LCD^ in the presence of sHsps. Data plotted as mean ± SD (*n* ≥ 3 technical replicates from two independent experiments). Data analyzed by one‐way ANOVA with Dunnett's post‐hoc test with comparison to NaCl control (****p < 0.0001).

As the sHsp HspB1 regulates the cytoplasmic condensation of TDP‐43 (Lu et al., 2022), we investigated how HspB1 modifies TDP‐43 phase separation in vitro and determined whether its homolog HspB5 also influences TDP‐43 condensation. Considering we previously observed that HspB1^3D^ and HspB5^3D^ are more effective at preventing TDP‐43^LCD^ fibrillation (Figure 3), we first assessed if wild‐type and phosphomimetic forms of HspB1 and HspB5 would affect TDP‐43^LCD^ condensation using light scatter and DIC imaging. We observed that the addition of HspB1^WT^ promoted TDP‐43^LCD^ condensation to levels similar to 200 mM NaCl addition (no salt added with chaperone), whereas HspB1^3D^ significantly increased TDP‐43^LCD^ condensate formation even in the absence of NaCl (Figure 6a), indicating that HspB1^3D^ promotes TDP‐43^LCD^ phase separation under these conditions. These condensates appeared larger and more numerous than those formed in the presence of NaCl.

Similarly, the wild‐type (HspB5^WT^) and phosphomimetic (HspB5^3D^) forms of HspB5 were evaluated for their impact upon TDP‐43^LCD^ condensate formation, noting that phosphomimicking mutations have a less pronounced effect upon its oligomeric distribution (Johnson et al., 2009; Kato et al., 1997; Li, Chiang, et al., 2018). In contrast to the ability of HspB1^WT^ to potentiate TDP‐43^LCD^ condensation, few condensates were observed when TDP‐43^LCD^ was incubated with HspB5^WT^, indicating that HspB5 does not promote condensation of TDP‐43^LCD^ under these conditions (Figure 6a). Similarly, few condensates were observed when TDP‐43^LCD^ was incubated with HspB5^3D^ (Figure 6a), indicating that the enhanced negative charge imparted by phosphomimicking mutations does not drive HspB1 to promote TDP‐43^LCD^ condensate formation. Quantification of these differences via a kinetic light scattering assay revealed significantly greater turbidity at all wavelengths measured when TDP‐43^LCD^ was incubated with HspB1^3D^ compared to all other experimental conditions (Figure 6b,c). These findings suggest that, despite their sequence and structural similarities, HspB1 and HspB5 interact differently with TDP‐43^LCD^ under conditions that promote its phase separation.

### 2.7 The ACDs of HspB1 and HspB5 do not significantly impact TDP‐43^LCD^ condensation

**FIGURE 7:**
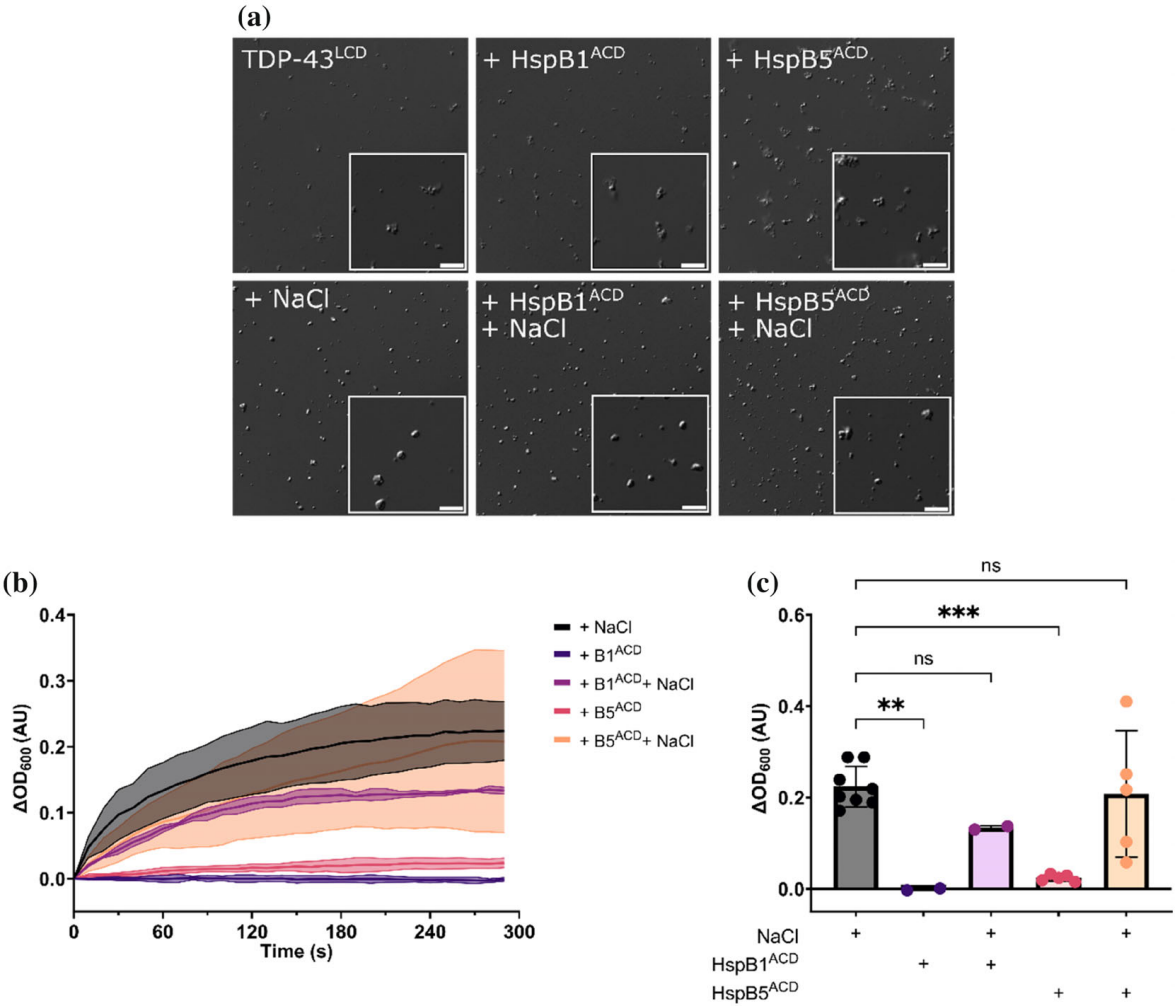
HspB1^ACD^ and HspB5^ACD^ do not significantly impact condensation of TDP‐43^LCD^. TDP‐43^LCD^ (20 μM) in 20 mM MES (pH 6.0) was incubated with HspB1^ACD^ (20 μM) or HspB5^ACD^ (20 μM) in the presence or absence of NaCl (200 mM). (a) Differential interference contrast (DIC) confocal microscopy images of TDP‐43^LCD^ condensates formed in the presence of either chaperone. Scale bars represent 5 μm. (b,c) Quantification of the change in OD at 600 nm following mixing of TDP‐43^LCD^ with sHspse. (c) Change in OD as a function of time in the presence of ACDs. (c) Endpoint OD value for TDP‐43^LCD^ in the presence of ACDs. Data plotted as mean ± SD (*n* ≥ 2 technical replicates from two independent experiments). Data analyzed by one‐way ANOVA with Dunnett's post‐hoc test in comparison to NaCl control (** = *p* < 0.01, *** = *p* < 0.001).

We sought to ascertain whether the central α‐crystallin domain of HspB1 played a role in promoting the condensation of TDP‐43^LCD^. To do so, TDP‐43^LCD^ was incubated with HspB1^ACD^ or HspB5^ACD^, either in the absence or presence of NaCl (Figure 7). Incubation of TDP‐43^LCD^ with either HspB1^ACD^ or HspB5^ACD^ in the absence of NaCl resulted in the formation of small clusters of condensates that appeared more numerous than those formed by TDP‐43^LCD^ alone (Figure 7a). Despite this, both samples exhibited low endpoint OD compared to TDP‐43^LCD^ incubated with NaCl (Figure 7b,c). In the presence of NaCl, HspB1^ACD^ and HspB5^ACD^ did not significantly alter the appearance of TDP‐43^LCD^ condensate (Figure 7a), nor did they alter the endpoint OD of these samples (Figure 7b,c). Thus, neither HspB1^ACD^ nor HspB5^ACD^ significantly altered the condensation of TDP‐43^LCD^, consistent with them having a high degree of secondary structure that is generally considered incompatible with condensation. From these findings, we conclude that HspB1 promotes TDP‐43^LCD^ phase separation via interactions involving its disordered N‐ and/or C‐terminal regions.

### 2.8 HspB1 and HspB5 partition into TDP‐43^LCD^ condensates

**FIGURE 8:**
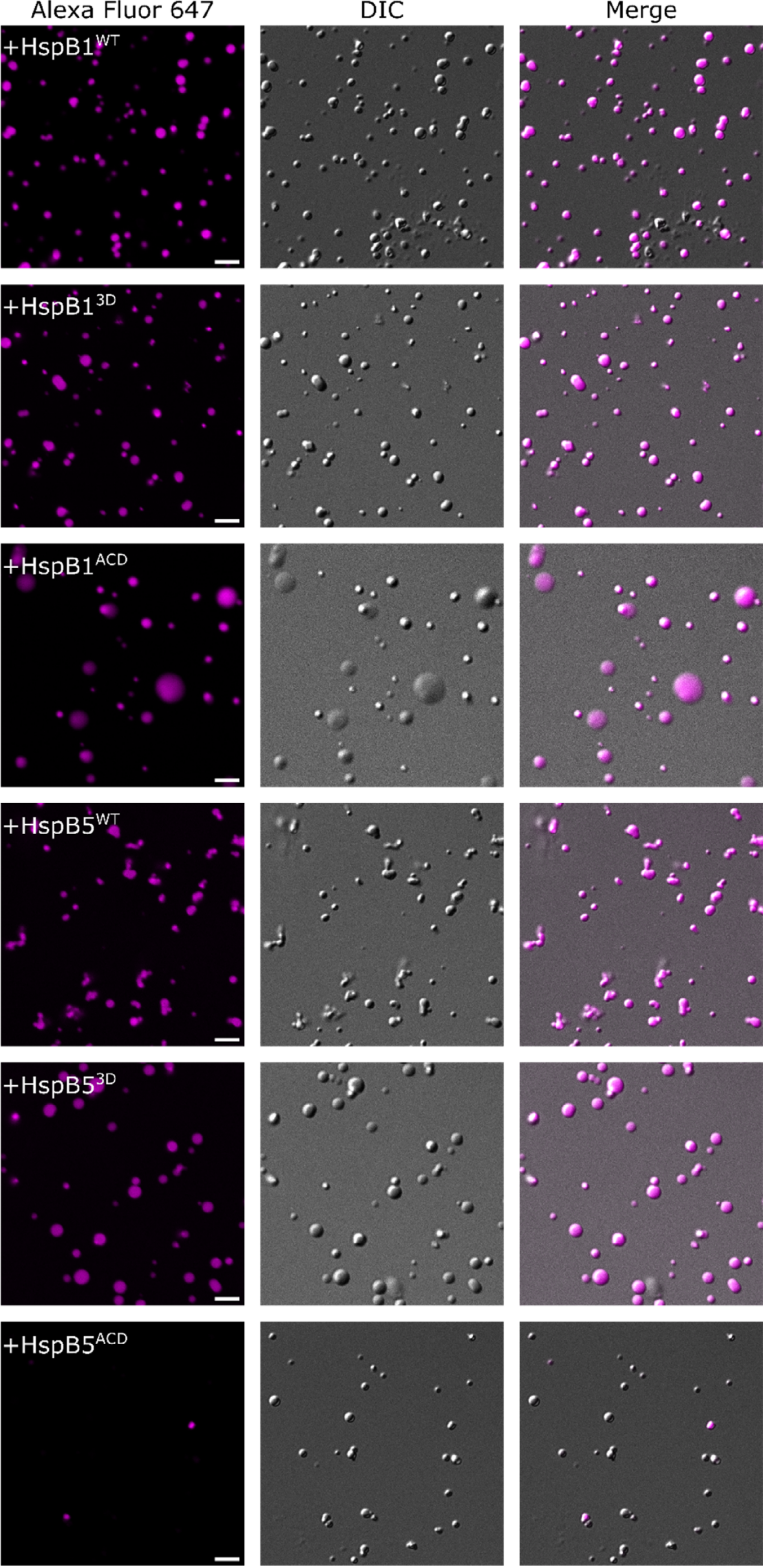
HspB1 and HspB5 localize within TDP‐43^LCD^ condensates. TDP‐43^LCD^ (20 μM) was induced to phase separate in the presence of Alexa Fluor 647‐labeled forms of HspB1^WT^, HspB1^3D^, HspB1^ACD^, HspB5^WT^, HspB5^3D^, or HspB5^ACD^. Images were taken using a Leica TCS SP5 laser scanning confocal microscope equipped with a 40×/0.75 NA water immersion objective.

HspB1 has been reported to partition within cytoplasmic TDP‐43 condensates, where it interacts with the RNA binding domain and LCD of TDP‐43 (Lu et al., 2022). Therefore, we investigated whether the effects of HspB1 and HspB5 upon TDP‐43^LCD^ condensation correlates with their incorporation into condensates. To this end, we incubated TDP‐43^LCD^ with Alexa Fluor 647‐labeled full‐length or ACD isoforms of the sHsps to investigate their possible localization in these condensates. We avoided using dual labeled proteins in these experiments to avoid potential quenching artifacts in the condensed phase (Hubatsch et al., 2021; McCall et al., 2025) Expectedly, we observed that wild‐type and phosphomimetic forms of HspB1 and HspB5, as well as their core ACDs, localized within TDP‐43^LCD^ condensates (Figure 8). In contrast, the non‐chaperone control protein was excluded from TDP‐43^LCD^ condensates (Figure S4). As such, the localization of these chaperones within condensates was due to specific interactions between each chaperone isoform and TDP‐43^LCD^ (Figures S2 and S3). These findings indicate that, in addition to inhibiting the fibrillar aggregation of TDP‐43^LCD^, these chaperones may also modify the dynamic properties of TDP‐43^LCD^ condensates.

### 2.9 Both HspB1^3D^ and HspB5^3D^ enhance the dynamic nature of TDP‐43^LCD^ condensates

**FIGURE 9:**
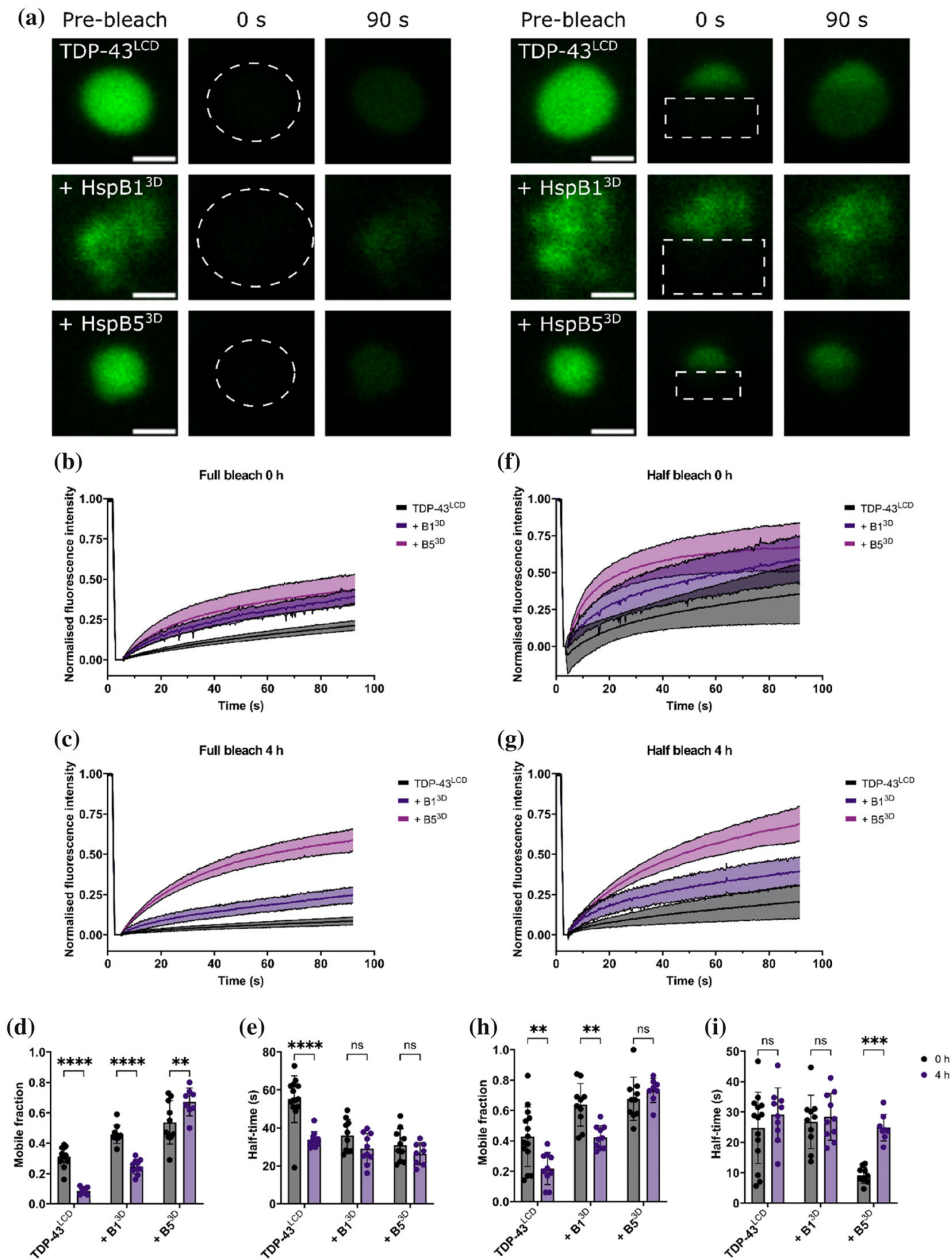
HspB1 and HspB5 differentially alter the dynamic properties of TDP‐43^LCD^ condensates. Cy5‐labeled TDP‐43^LCD^ was induced to phase separate in the presence of HspB1^3D^ or HspB5^3D^ and evaluated by FRAP. The fluorescence recovery of fully bleached or half‐bleached TDP‐43^LCD^ condensates was monitored over ~90 s and analyzed using EasyFRAP to calculate the mobile fraction and half‐time to recovery. (a) Example images showing fluorescence recovery of photobleached TDP‐43^LCD^ condensates. Left side shows fully bleached condensates. Right shows half‐bleached condensates. Scale bars represent 2 μm. (b,c) FRAP curves for fully bleached TDP‐43^LCD^ condensates in the absence or presence of HspB1^3D^ or HspB5^3D^ at (b) 0 h and (c) 4 h. (d) Mobile fraction of fully bleached condensates at 0 and 4 h. (e) Half‐time to recovery of fully bleached condensates at 0 and 4 h. (f, g) FRAP curve for half‐bleached TDP‐43^LCD^ condensates in the absence or presence of HspB1^3D^ or HspB5^3D^ at (f) 0 h and (g) 4 h. (h) Mobile fraction of half‐bleached TDP‐43^LCD^ condensates at 0 and 4 h (i) Half‐time to recovery of half‐bleached TDP‐43^LCD^ condensates at 0 and 4 h. Data plotted as mean ± SD (b–i) (*n* ≥ 8 technical replicates from at least two independent experiments). Data analyzed by (d, e, h, i) two‐way ANOVA with Šidak's post (**p < 0.01, ***p < 0.001, ****p < 0.0001).

In the cell, stress‐inducing conditions promote the cytoplasmic condensation and aggregation of TDP‐43 characteristic of ALS (Lu et al., 2022). These same conditions also promote the phosphorylation of sHsps such as HspB1 and HspB5 (Janowska et al., 2019). Therefore, we sought to evaluate the specific effect of HspB1^3D^ and HspB5^3D^ upon TDP‐43^LCD^ condensation in a simplified purified protein model using Cy5‐labeled TDP‐43^LCD^. To this end, we performed fluorescence recovery after photobleaching (FRAP) (Axelrod et al., 1976). In these experiments, the entire area of a condensate was bleached to evaluate the movement of TDP‐43^LCD^ protein into condensates. By plotting the fluorescence recovery over time, we derived two key parameters: (i) the mobile fraction, which is the fraction of TDP‐43^LCD^ capable of exchange and (ii) half‐time, which is the time taken to reach half‐maximal fluorescence recovery and is an indicator of the speed at which TDP‐43^LCD^ undergoes exchange. Here, we observed distinct differences in the dynamic exchange of TDP‐43^LCD^ within condensates and between the condensed and dilute phases when in the presence or absence of HspB1^3D^ and HspB5^WT^ (Figure 9b–d). In the absence of either sHsp, fully bleached TDP‐43^LCD^ condensates exhibited poor fluorescence recovery (Figure 9b). In the presence of HspB1^3D^ or HspB5^3D^, fully bleached TDP‐43^LCD^ condensates exhibited far greater fluorescence recovery (Figure 9c), indicative of enhanced exchange with the surrounding dilute phase. After 4 h, the fluorescence recovery of fully bleached TDP‐43^LCD^ condensates was reduced, with a significantly lower mobile fraction and shorter half‐time to recovery (Figure 9c), suggesting that these condensates had become less dynamic and more solid‐like. Similarly, condensates in the presence of HspB1^3D^ also exhibited reduced recovery after 4 h, with a lower mobile fraction and a similar half‐time to recovery (Figure 9c–e). In contrast, condensates formed in the presence of HspB5^3D^ maintained a high level of fluorescence recovery after 4 h, maintaining a high mobile fraction and a similar half‐time to recovery. Together, these findings indicate that HspB1^3D^ and HspB5^3D^ modify the mobility of TDP‐43^LCD^ between the condensed and dispersed phases.

We also evaluated the dynamic exchange of TDP‐43^LCD^ within the condensed phase via FRAP. To do so, approximately half of a condensate was bleached to evaluate the movement of TDP‐43^LCD^ protein within individual condensates. The internal dynamics of TDP‐43^LCD^ were relatively low and were enhanced in the presence of either HspB1^3D^ or HspB5^3D^ (Figure 9f). While the recovery of half‐bleached condensates was reduced after 4 h both in the presence and absence of HspB1^3D^, exhibiting a reduced mobile fraction in both instances (Figure 9g,h). In contrast, their dynamics were maintained in the presence of HspB5^3D^ (Figure 9f). Indeed, these condensates retained a similar mobile fraction at 0 and 4 h, though they had a longer half‐time to recovery after 4 h (Figure 9g,h), suggesting that HspB5^3D^ maintained the internal dynamics of TDP‐43^LCD^ condensates.

## 3 DISCUSSION

While condensates and amyloids can arise via distinct protein interactions, aggregation via condensation is primarily driven by protein regions that can engage in multimodal interactions (Vendruscolo & Fuxreiter, 2022). Invariably, these regions are intrinsically disordered and, thus, sample conformationally heterogeneous states. Therefore, the cellular mechanisms that maintain the dynamic state of protein condensates will differ from those that maintain proteins in a soluble state. The capacity of sHsps to inhibit the assembly of amyloid‐like fibrils is well established (Carra et al., 2017; Janowska et al., 2019; Nakamoto & Vigh, 2007), however, much less is known about how sHsps engage with potential client proteins during condensation. In this study, we show that HspB1 and HspB5 differentially interact with TDP‐43^LCD^ during its condensation and aggregation. Notably, we demonstrate that HspB1 promotes the spontaneous condensation of TDP‐43^LCD^, further emphasizing the distinct interactions between different sHsps and the same client protein.

The functional differences between HspB1 and HspB5 may be explained by sequence (and structural) differences that exist throughout their amino acid sequences, particularly in the variable N‐ and C‐terminal domains. Although full‐length isoforms of HspB1 and HspB5 exhibit the generic ability to inhibit the fibrillar aggregation of TDP‐43^LCD^, similar examination of their central ACDs revealed opposite effects upon TDP‐43^LCD^ aggregation. Furthermore, while full length HspB1 promoted TDP‐43^LCD^ condensation and enhanced the dynamic properties of TDP‐43^LCD^ condensates, the isolated ACD did not significantly impact TDP‐43^LCD^ condensation despite it (and the ACD of HspB5) localizing within condensates. Despite being comparatively less potent than their full‐length isoforms, the isolated ACDs of HspB1 and HspB5 were both found to specifically interact with small oligomers of TDP‐43^LCD^, indicating a role for this region in client binding. These findings indicate that the divergences in the functional interactions of HspB1 and HspB5 with TDP‐43^LCD^ are primarily derived from differences in the N‐ and/or C‐terminal regions, which share 38% and 37% sequence similarity, respectively. Indeed, this is consistent with previous work demonstrating that binding of various clients by HspB1 and HspB5 involves distinct regions primarily situated in the variable N‐ and/or C‐terminal domains that flank the central α‐crystallin domain (Cox et al., 2016; Cox & Ecroyd, 2017; Freilich et al., 2018; Hochberg et al., 2014; Mainz et al., 2015; McDonald et al., 2012; Selig et al., 2020). Together, these findings suggest that HspB1 and HspB5 engage with TDP‐43 via multivalent interactions.

Although this study did not examine which regions in TDP‐43^LCD^ are bound by HspB1 and HspB5 during condensation and fibrillation, previous work has revealed structural features within TDP‐43^LCD^ that govern these behaviors. Condensation of TDP‐43^LCD^ involves aromatic amino acids flanked by glycine and serine residues, which contribute to condensation through intermolecular π‐stacking and cation‐π interactions (Conicella et al., 2016; Pantoja‐Uceda et al., 2021). Interestingly, similar aromatic motifs are present in the disordered N‐terminal region of HspB1, suggesting that TDP‐43^LCD^ condensation promoted by HspB1 may occur via intermolecular interactions involving these motifs on both proteins. Furthermore, TDP‐43^LCD^ harbors a transient α‐helical region that is crucial for condensation and undergoes structural transformation to a β‐sheet during fibrillation, forming the fibril core (Arseni et al., 2022; Conicella et al., 2016). Given their potent ability to recognize and bind misfolded proteins, it is likely that HspB1 and HspB5 bind this amyloidogenic core region within TDP‐43^LCD^ to prevent its assembly into fibrils. Further investigation of the molecular mechanisms of these interactions may guide the development of therapeutics against TDP‐43 proteinopathies.

The oligomeric state of sHsps influences their chaperone activity (Aquilina et al., 2004; Aquilina et al., 2013; Ecroyd et al., 2007; Jovcevski et al., 2015; Peschek et al., 2013) and this is altered by stress‐induced phosphorylation of serine residues that reside within the N‐terminal domain of both HspB1 and HspB5 (Ahmad et al., 2008; Arrigo & Gibert, 2013; Boelens, 2020; Ecroyd et al., 2007; Ito et al., 2001; Peschek et al., 2013). Mutations that mimic phosphorylation events (i.e., in the case of HspB1, at serine residues 15, 78, and 82) cause HspB1 to dissociate from large oligomers to dimers (Jovcevski et al., 2015). Similar mutations in HspB5 (at serine residues 19, 45, and 59) have a far less pronounced effect upon its oligomeric distribution. The increase in chaperone activity because of mutations that mimic phosphorylation was strongly reflected in our findings, as the phosphomimetic mutant, HspB1^3D^, was more effective at inhibiting TDP‐43^LCD^ aggregation than the wild‐type isoform. Notably, the cellular conditions that promote sHsp phosphorylation are the same conditions that promote TDP‐43 condensation and aggregation (Lu et al., 2022), indicating that the consequent dissociation of HspB1 into dimers functions to enhance its affinity for misfolded TDP‐43. In contrast, phosphomimetic HspB5^3D^ had a similar impact upon TDP‐43^LCD^ fibrillation as its wild‐type counterpart, reflective of a similar oligomeric substructure. Notably, the enhanced dynamics of TDP‐43^LCD^ in the presence of phosphomimetic HspB1^3D^ and HspB5^3D^ are unlikely to be driven by differences in the net charge of the sHsps, as only HspB5^3D^ maintained the dynamics of TDP‐43^LCD^ condensates over time, thereby antagonizing TDP‐43^LCD^ phase transitions. The distinct effects upon TDP‐43^LCD^ fibrillation and condensation suggest that HspB1 and HspB5 interact differently with TDP‐43^LCD^.

We made specific comparisons between HspB1 and HspB5 here in relation to the aggregation and phase transition of the TDP‐43^LCD^, finding chaperone‐specific effects. An area we did not explore involves the more biologically relevant formation of sHsp hetero‐oligomers composed of multiple members of the sHSP family (Den Engelsman et al., 2009; Mymrikov et al., 2020; Tedesco et al., 2022). Hetero‐oligomers composed of sHsps are considered to have chaperone‐specific effects that rely upon their underlying sHsp stoichiometries. Of particular interest in this area is the relationship between HspB2 and HspB3. An excess of HspB2 compared to HspB3 promotes condensation of the former in the nucleus (Joosten et al., 2023; Morelli et al., 2017), whereas a higher ratio of HspB3 to HspB2 results in solid aggregates. A difficulty in this area is knowing the composition of hetero‐oligomers, ratios of hetero‐ and homo‐oligomers in poly‐disperse solutions, and the proportion of dimers (which are considered to be the most chaperone active form [Jovcevski et al., 2015; Tedesco et al., 2022]). Future work should explore this area to better understand how the heterogeneous nature of sHsps contributes to their roles in phase transitions and aggregation of TDP‐43 and other proteins.

Insights from patient‐derived tissue also reveal the therapeutic potential of HspB1 and HspB5. Proteomics analysis of sporadic ALS brain and spinal cord tissue has revealed that the levels of HspB1 and HspB5 are negatively correlated with the severity of TDP‐43 pathology (Guise et al., 2024). Furthermore, transcriptomic analysis of iPSC‐derived motor neurons from sporadic ALS patients has shown that HspB1, but not HspB5, is upregulated during periods of oxidative stress in which TDP‐43 assembles into cytoplasmic condensates (Lu et al., 2022). In contrast, the absence of HspB5 in these analyses suggests that HspB5 may fail to be transcriptionally upregulated by the onset of disease pathology in ALS motor neurons, which is reflective of the broader deficiency of proteostasis that is characteristic of ALS (Yerbury et al., 2020). The promising results presented in this study demonstrate the potent ability of HspB5 to delay TDP‐43^LCD^ aggregation and maintain TDP‐43^LCD^ condensate dynamics, which suggests that HspB5, along with HspB1, may be a viable therapeutic target for delaying ALS disease progression. Indeed, overexpression of HspB1 in the motor neurons of transgenic SOD1 G93A mice was found to inhibit disease progression (Sharp et al., 2008), demonstrating its potential as a broad protectant against protein aggregation in ALS.

Protein condensation and fibrillation are both potential, sometimes overlapping, pathways that may lead to the formation of protein inclusions in neurodegenerative diseases. Therefore, characterizing the mechanistic differences in these processes and the cellular systems that regulate them will be instrumental in our understanding of diseases such as ALS and FTLD‐TDP. Our findings that TDP‐43 condensation and fibrillation are differentially regulated by chaperones from the same class suggest that other proteins implicated in neurodegenerative diseases may also be regulated in distinct manners. As such, the design of therapeutics must reflect the understanding that condensation and fibrillation are both potential pathways that can lead to the formation of protein inclusions. In conclusion, this study demonstrates the potent ability of HspB1 and HspB5 to modify the condensation and aggregation of TDP‐43. The insights provided by these findings will facilitate the advancement of therapeutics for the treatment of ALS and other neurodegenerative diseases.

## 4 MATERIALS AND METHODS

### 4.1 Preparation of bacterial expression vectors

The pJ411 bacterial expression vector for the expression of the TDP‐43 LCD (TDP‐43^LCD^) was a kind gift from Professor N. Fawzi (Brown University, Providence, RI; Addgene #98669). The pET3a and pET24a bacterial expression vectors containing the human *HSPB1* (HspB1) and *HSPB5* (HspB5) were used for expression of recombinant wild‐type proteins (Ito et al., 2001). A variant of HspB1 designed to mimic phosphorylation at serines 15, 78, and 82 was generated by site‐directed mutagenesis of serine residues to aspartic acid to produce HspB1^3D^ (Ito et al., 2001). The pET28 bacterial expression vectors for the expression of the core domains of HspB1 (residues 86–169, HspB1^ACD^) and HspB5 (residues 68–162, HspB5^ACD^) were a kind gift from Professor A. Laganowsky (Texas A&M Health Science Center; Laganowsky et al., 2010). Details of all bacterial expression plasmids are shown in Table S1. All recombinant proteins were transformed and expressed in *Escherichia coli* BL21(DE3) cells and purified as previously described (Selig et al., 2020; Sharp et al., 2008) or using a modification of a previously published protocol (Conicella et al., 2016). Protein molecular weight standards used in gel electrophoresis were Protein Precision Plus Dual Color obtained from Bio‐Rad Laboratories. All other chemicals, including ThT, were obtained from Sigma Aldrich, unless otherwise stated. Protein concentrations were determined using a Nanodrop 2000c spectrophotometer (ThermoFisher Scientific, Waltham, MA) based upon extinction coefficient values of 19,480 M^−1^ cm^−1^ for TDP‐43^LCD^, 37,587 M^−1^ cm^−1^ for HspB1, 8605 M^−1^ cm^−1^ for HspB1^ACD^, 16,732 M^−1^ cm^−1^ for HspB5, and 1490 M^−1^ cm^−1^ for HspB5^ACD^ as calculated using the ExPASy ProtParam tool (Gasteiger et al., 2005).

### 4.2 Expression and purification of TDP‐43^LCD^

The basis for the protocols describing TDP‐43^LCD^ expression and purification has been published previously (Conicella et al., 2016). In this study, the protocol was modified for the generation of non‐isotopically labeled TDP‐43^LCD^ protein from inclusion bodies. Initial 10 mL LB media starter cultures containing 50 μg/mL kanamycin were inoculated with a single colony of transformed pJ411 TDP‐43_LCD BL21(DE3) *E. coli* and incubated overnight with orbital shaking at 180 rpm at 37°C. Each 10 mL overnight starter culture was used to inoculate 1 L of 2YT media containing 50 μg/mL kanamycin. Main cultures were grown at 37°C with orbital shaking at 200 rpm until an OD_600_ of 0.6 was reached, at which point isopropyl β‐D‐1 thiogalactopyranoside (IPTG) was added to a final concentration of 0.5 mM. After induction, cultures were incubated for 6 h at 37°C with orbital shaking at 200 rpm. Bacteria were then harvested by centrifugation at 6000*g* for 10 min at 4°C and stored at −20°C overnight. Bacterial pellets were resuspended in 50 mM Tris‐base, 500 mM NaCl, 20 mM imidazole (pH 8.0) (25 mL per liter of main culture) with the addition of one tablet of EDTA‐free protease inhibitor and lysozyme and phenymethylsulfonyl fluoride (PMSF) to final concentrations of 0.5 mg/mL and 2 mM, respectively. Cells were incubated for 30 min with gentle rocking at room temperature before being lysed using a sonicator at 10% amplitude for 20 s, five times. Insoluble material containing TDP‐43^LCD^ was pelleted by centrifugation at 40000*g* for 20 min at 4°C and resuspended in 50 mM Tris‐base, 500 mM NaCl, 20 mM imidazole, 2% (v/v) Triton X‐100 (pH 8.0) (10 mL per pellet). This was repeated twice more, before being resuspended in 50 mM Tris‐base, 1 M NaCl, 20 mM imidazole (pH 8.0) (10 mL per pellet) and incubated for 30 min at 4°C with gentle rocking. The lysate was pelleted by centrifugation at 40000*g* for 20 min at 4°C. The pellet was resuspended in filtered and degassed 20 mM Tris‐base, 500 mM NaCl, 20 mM imidazole, 8 M urea (pH 8.0) (25 mL per pellet) and centrifuged at 40000*g* for 20 min at 4°C. The supernatant was decanted and filtered using a 0.22 μm filter and either subjected to nickel‐affinity chromatography or stored at −80°C for subsequent purification.

Filtered TDP‐43^LCD^ samples were injected onto a HisTrap™ column equilibrated in binding buffer containing 20 mM Tris‐base, 500 mM NaCl, 20 mM imidazole, 8 M urea (pH 8.0) at a flow rate of 2 mL/min. Once the sample had completely run through, the column was washed with binding buffer at a flow rate of 3 mL/min to remove unbound proteins from the column. The protein was eluted with a gradient of 20–500 mM imidazole at a flow rate of 2.5 mL/min and collected in fractions of 10 mL volume. Fractions containing pure TDP‐43^LCD^ were determined by SDS‐PAGE, pooled, and aliquots frozen at −80°C for later use.

### 4.3 Expression and purification of sHsps and their ACDs

The expression and purification of HspB1^WT^, HspB1^3D^, HspB1^ACD^, HspB5^WT^, and HspB5^ACD^ were performed as described previously (Aquilina et al., 2013).

### 4.4 SDS‐PAGE analysis of TDP‐43^LCD^ purity

SDS‐PAGE analysis was used to evaluate the expression and purity of TDP‐43^LCD^ protein. A 10% (v/v) acrylamide resolving gel was poured into a MINI PROTEAN® system (Bio‐Rad, USA) and overlayed with 70% ethanol. Resolving gels were allowed to set for 20 min, after which the overlayed 70% ethanol was removed by absorption into paper towel. A 4% (v/v) stacking gel mix was then overlayed, and a gel comb inserted to form wells. The stacking gel was allowed to set for 20 min. Protein samples were mixed with an appropriate volume of reducing SDS‐PAGE sample buffer (final concentrations: 10% (v/v) glycerol, 2% SDS, 0.1% bromophenol blue, 1.25% (v/v) β‐mercaptoethanol, 62.5 mM Tris–HCl pH 6.8), and boiled for 5 min, after which samples were loaded into wells. The gel was then run at 150 V in 1× SDS running buffer (192 mM glycine, 3.5 mM SDS, 25 mM Tris) for 1 h. Afterwards, the gel was microwaved in MilliQ water for 30 s at maximum power and incubated at room temperature for 5 min with gentle rocking. This was repeated once more with fresh MilliQ water, before protein bands were visualized by incubation in Coomassie Brilliant Blue G‐250 stain solution (Sigma Aldrich) for 15–30 min at room temperature with gentle rocking. Gels were destained by incubating them in MilliQ water for 30 min at room temperature with gentle rocking until proteins were clearly resolved. Protein bands of interest were identified using Precision Plus Protein™ dual color molecular weight markers (Bio‐Rad, USA).

### 4.5 Buffer exchange of proteins

Purified proteins were buffer exchanged into 20 mM MES (pH 6.0) prior to all analyses using Zeba Spin 7000 MWCO desalting columns (Sigma Aldrich). Briefly, spin columns were centrifuged at 1500*g* for 1 min to remove the storage solution. Columns were equilibrated by adding 20 mM MES (pH 6.0) and centrifuging at 1500*g* for 1 min. This was repeated 4 times. Samples were aliquoted into the spin columns and buffer exchanged into 20 mM MES (pH 6.0) via centrifugation at 1500*g* for 2 min (for TDP‐43^LCD^) or 30 s (HspB1 and HspB5) into a low‐protein binding microcentrifuge tube. Samples were immediately centrifuged at 16,300*g* for 5 min to pellet any insoluble material. The supernatant was transferred to a fresh low‐protein binding microcentrifuge tube and diluted to the desired concentration.

### 4.6 Thioflavin‐T assay

The formation of TDP‐43^LCD^ aggregates was monitored using an in situ thioflavin‐T (ThT) binding assay. Briefly, purified TDP‐43^LCD^ protein was buffer exchanged from 8 M urea into 20 mM MES (pH 6.0) as described above. The protein was immediately centrifuged to pellet any particulate material and the supernatant was transferred to a fresh low‐protein binding microfuge tube and its concentration determined. Varying concentrations (50, 20, 10, 5, and 2.5 μM) of TDP‐43^LCD^ were incubated with 25 μM ThT in 20 mM MES (pH 6.0) at room temperature and loaded into a clear‐bottomed low‐protein binding 384‐well plate (Greiner, Germany). The plate was incubated in a PolarStar Omega Plate Reader (BMG Labtechnologies, Australia) at 37°C for 30 min before being sealed with adhesive film. Excitation and emission filters were set at 440 and 490 nm, respectively. The plate underwent double orbital shaking at 200 rpm for 30 s at the beginning of a 350 s cycle for at least 180 cycles, totalling ~72 h.

The formation of TDP‐43^LCD^ aggregates in the presence of sHsps was also monitored using an in situ ThT binding assay. Briefly, 10 μM TDP‐43^LCD^ was incubated with varying concentrations (50, 10, 2, 1, or 0.2 μM) of either HspB1 or HspB5 with 25 μM ThT in 20 mM MES (pH 6.0) at room temperature. The reaction mixtures were loaded into a clear‐bottomed low‐protein binding 384‐well plate (Greiner, Germany) and incubated in a PolarStar Omega Plate Reader (BMG Lab Technologies, Australia) at 37°C for 30 min before being sealed with adhesive film. The plate underwent double orbital shaking at 200 rpm for 30 s at the beginning of a 350 s cycle for at least 742 cycles, with ThT fluorescence measured by excitation at 450 nm and emission read at 480 nm using the bottom optic of the plate reader.

The kinetics of aggregation were analyzed by deriving the length of the lag‐phase and fibril elongation rate from the equation for the Boltzmann sigmoidal curve. The main parameters used in this equation are final fluorescence (*F*_*f*_), initial fluorescence (*F*_*i*_), time to half maximal fluorescence (*t*_*50*_), and the slope of the curve (*k*). The following equations have been described previously (Nielsen et al., 2001).

The equation of the Boltzmann‐sigmoidal curve is given as:

$$F=F_{i}+\frac{\left(F_{f}-F_{i}\right)}{1+e^{\frac{\left(t_{50}-t\right)}{k}}}$$

The slope of the line at any point is:

$$\frac{\mathrm{dF}}{\mathrm{dt}}=\frac{\frac{\left(F_{f}-F_{i}\right)}{k}\times e^{\frac{\left(t_{50}-t\right)}{k}}}{{\left(1+e^{\frac{\left(t_{50}-t\right)}{k}}\right)}^{2}}$$

When *t* = *t*_*50*_ (i.e., the inflection point, when the elongation rate is maximal), the elongation rate is:

$$\frac{\mathrm{dF}}{\mathrm{dt}}=\frac{\frac{\left(F_{f}-F_{i}\right)}{k}\times e^{0}}{{\left(1+e^{0}\right)}^{2}}$$

$$=\frac{\left(F_{f}-F_{f}\right)}{4\times k}$$

The lag phase is when:

$$\left\{\frac{\left(F_{f}-F_{f}\right)}{4\times k}\right\}\times t_{50}+b=F_{i}$$

To solve for *b*, at *t*_*50*_ (i.e., the midpoint between *F*_*f*_ and *F*_*i*_):

$$F_{t_{50}}=\frac{\left(F_{f}-F_{i}\right)}{2}$$

$$b=\left\{\frac{\left(F_{f}-F_{i}\right)}{4\times k}\right\}\times t_{\mathrm{lag}}+\left\{\frac{\left(F_{f}-F_{i}\right)}{2}\right\}-\left\{\frac{\left(F_{f}-F_{i}\right)}{4\times k}\right\}\times t_{50}$$

Solving for *t*_*lag*_:

$$4\times k\times F_{i}=\begin{array}{l} \left\{\left(F_{f}-F_{i}\right)\times t_{\mathrm{lag}}\right\}+\left\{2\times k\left(F_{f}-F_{i}\right)\right\}\\ -\;\left\{\left(F_{f}-F_{i}\right)\times t_{50}\right\} \end{array}$$

$$\left(F_{f}-F_{i}\right)\times t_{\mathrm{lag}}=\begin{array}{l} 4\times k\times F_{i}+\left\{\left(F_{f}-F_{i}\right)\times t_{50}\right\}\\ -\;\left\{2\times k\left(F_{f}-F_{i}\right)\right\} \end{array}$$

$$t_{\mathrm{lag}}=\frac{4\times k\times F_{i}}{\left(F_{f}-F_{i}\right)}+t_{50}-\left\{2\times k\right\}$$

The relative efficacy of each sHsp to inhibit the formation of TDP‐43^LCD^ fibrils was determined by calculating the protection conferred by each sHsp according to the difference in maximal ThT fluorescence in the absence and presence of the chaperone using the equation,

$$\%\text{Protection}=\frac{∆I-∆I_{\text{chaperone}}}{∆I\times 100}$$

Wherein Δ*I* and Δ*I*_chaperone_ correspond to the change in ThT fluorescence of TDP‐43^LCD^ in the absence and presence of the sHsp, respectively.

### 4.7 Kaplan–Meier analysis of TDP‐43^LCD^ fibrillation in the presence of sHsps

Kaplan–Meier analysis was performed as previously described (Lu et al., 2022). We chose to display the data in this way to account for technical replicates in which aggregation was fully suppressed over the course of the experiment. Plots were generated using GraphPad Prism software. To construct the plots from the kinetic data, we used values of *t*_lag_ as the parameter that indicated that an aggregation event had occurred in a particular well on the basis that an increase in ThT fluorescence reflects its binding to a fibril. Each aggregation event was assigned the value “1.” Those wells that did not exhibit any significant increase in ThT were assigned the value “0” to indicate the absence of an aggregation event across the time of the assay. The statistical difference between the Kaplan–Meier plots of each well in comparison to TDP‐43^LCD^ alone was determined using the log‐rank (Mantel‐Cox) algorithm at 95% confidence interval. Hazard ratios were calculated with the log‐rank (Mantel‐Cox) method using GraphPad Prism 10 software. The hazard ratio for TDP‐43^LCD^ in different ratios of sHsp was calculated relative to TDP‐43^LCD^ alone. To calculate the mean time to failure, Kaplan–Meier plots were fit with a Boltzmann sigmoidal curve, from which values of *t*_lag_ were derived and assigned as the mean time to failure.

### 4.8 Transmission electron microscopy

Negative stain transmission electron microscopy (TEM) samples were prepared by applying 5 μL of fibril samples to 400 mesh carbon‐coated copper grids (ProSciTech, Queensland, Australia) and incubating on the grid for 30 s. The grids were blotted dry and washed twice with 10 μL filtered water and blotted dry each time before being stained with 2% uranyl acetate for 30 s, blotted dry, and allowed to dry for 2 min. Imaging was performed on a T‐12 (FEI/Thermo Fisher Scientific) electron microscope.

### 4.9 Kinetic light scattering assay

To semi‐quantify TDP‐43^LCD^ phase separation, 20 μL of 40 μM TDP‐43^LCD^ was pipetted into a single well of a clear 384‐well plate containing 20 μL of 20 mM MES pH 6.0 containing 0–1 M NaCl at 100 mM increments, such that the final concentration of protein and NaCl was half of its original concentration. The scattering of light caused by particles including phase‐separated TDP‐43^LCD^ was then measured by OD at 340, 400, and 600 nm every 10 s for 30 cycles using a SpectroStar Plate Reader (BMG Labtechnologies, Melbourne, Australia). Assays were conducted using *n* = 5 biological replicates. At the end of each assay, data for the change in OD were plotted using GraphPad Prism version 10 (Graphpad Software Inc., San Diego, CA). The final data point for each well was plotted to determine the total change in OD (ΔOD) across the time course of the assay.

### 4.10 Confocal microscopy

To visually assess the extent of TDP‐43^LCD^ phase separation, condensates were imaged using differential interference contrast (DIC) confocal microscopy. Condensates were prepared by incubating 50 μL of 40 μM TDP‐43^LCD^ for 1 h with an equal volume of NaCl in 20 mM MES (pH 6.0) at concentrations varying from 0 to 1 M in 100 mM increments, such that the protein and NaCl were diluted to half the original concentration. Additionally, condensates were prepared by incubating with an equal volume of 40 μM HspB1^3D^ in 20 mM MES (pH 6.0) or an equal volume of 40 μM HspB5^WT^ in 20 mM MES (pH 6.0) containing 200 mM NaCl. After incubation, 5 μL aliquots were placed on a glass slide with SecureSeal™ adhesive imaging spacers (Thermo Fisher Scientific), which was then sealed with a cover slip. Images were captured using a 63× NA1.2 water immersion objective on a Leica SP5 laser scanning confocal microscope.

### 4.11 Quantification of phase separated TDP‐43^LCD^

To quantify the amount of TDP‐43^LCD^ within condensates, samples were incubated at room temperature for 1 h before being centrifuged at 16300*g* for 5 min to pellet phase separated material. Supernatants were transferred to a separate tube and their protein concentrations determined using a Nanodrop 2000c spectrophotometer. Data were plotted using GraphPad Prism version 10 (GraphPad Software Inc., San Diego, CA).

### 4.12 Fluorescent labeling of proteins

TDP‐43^LCD^ was dialysed into labeling buffer (20 mM Tris, 500 mM NaCl, 6 M guanidine‐HCl, pH 8.0). Following dialysis, 10 mM sodium bicarbonate was added prior to the addition of 8 M Cytiva Cy5 Mono NHS Ester (GEPA15101) (Cy5). Labeled TDP‐43^LCD^ was incubated on a rotary mixer overnight at 4°C, after which free Cy5 dye was removed using ZebaSpin™ 7 kDa molecular weight cutoff desalting columns (ThermoFisher). Aliquots of labeled TDP‐43^LCD^ were then stored at −80°C. The degree of labeling (DOL) was calculated as follows:

$$x=A_{280}-\left(\frac{A_{280\mathrm{DYE}}}{\mathrm{CF}}\right)\times \varepsilon 1$$

$$\text{Concentration}=\frac{x}{\mathrm{MW}}$$

$$y=\frac{A_{280\mathrm{DYE}}}{\text{Concentration}\times \varepsilon 2}$$

$$\text{Degree of labelling}\;\left(\mathrm{DOL}\right)=y\times 100$$

Wherein A_280_ = absorbance of protein measured at 280 nm, A_280DYE_ = absorbance of Cy3 measured at 280 nm, CF = correction factor of Cy5 (0.08), ε1 = extinction coefficient of TDP‐43^LCD^ (19,480 cm^−1^ M^−1^), MW = molecular weight of TDP‐43^LCD^ (17197.43 g mol^−1^), ε2 = extinction coefficient of Cy5.

Fluorescent labeling of HspB1^WT^, HspB1^3D^, HspB1^ACD^, HspB5^WT^, HspB5^3D^, and HspB5^ACD^ was performed as above but using Alexa Fluor 647NHS ester (Invitrogen A37573). The labeling efficiencies of proteins were as follows: TDP‐43^LCD^ (26%), HspB1^WT^ (150%), HspB1^3D^ (130%), HspB1^ACD^ (104%), HspB5^WT^ (30%), HspB5^3D^ (134%), and HspB5^ACD^ (5.5%).

### 4.13 Fluorescence recovery after photobleaching

To evaluate the effect of HspB1^3D^ and HspB5^3D^ upon the dynamic properties of TDP‐43^LCD^, condensates were evaluated via FRAP. Condensates were prepared as per section 2.2.9 with the addition of 1% Cy5‐labeled TDP‐43^LCD^. FRAP was performed using a Leica SP5 laser scanning confocal microscope equipped with a 63×/1.2 NA water immersion objective at 70% laser power. Bleaching was conducted at 100% laser transmission using the 488 nm line, while 6% laser transmission was using for imaging. PMT detectors were used and calibrated using digital gain to prevent signal oversaturation and 16‐bit images were obtained to improve signal dynamic range. Fluorescence recovery was monitored for ~90 s at intervals of ~0.171 s.

The fluorescence intensities for the bleached region, reference region (whole condensate), and background region (outside the condensate) were measured and analyzed using the program‐based tool, EasyFRAP (Koulouras et al., 2018). Data underwent full‐scale normalization, and mobile fraction and half‐time to recovery were derived from these curves. At least 8 image series were analyzed per sample to calculate the mean and standard deviation (SD). The mean mobile fraction and half‐time to recovery for each condition were plotted using Prism version 10 (GraphPad). Data is representative of at least two independent experiments.

### 4.14 Two‐color coincidence detection (TCCD)

Two‐color coincidence detection (TCCD) was performed as described previously (Chappard et al., 2023). Briefly, glass‐bottomed chamber slides (Ibidi) were blocked for 24 h in blocking buffer (10% (v/v) FCS, 2% (w/v) BSA, 0.1% (v/v) Triton X‐100, 1X PBS) before being washed with PBS. Following equilibration to 25°C, Cy5‐labeled TDP‐43^LCD^ or Alexa Fluor 647‐labeled HspB1^3D^, HspB1^ACD^, HspB5^WT^, or HspB5^ACD^ were diluted to 200 nM in 20 mM MES (pH 6.0) immediately prior to imaging. Two‐color coincidence detection (TCCD) was performed using a Leica SP8 FALCON confocal microscope (Leica, Wetzlar, Germany) using an 86×/1.20 water immersion objective using the white light laser (WLL) at 85% output and at a pulse rate of 80 MHz. Cy3‐labeled TDP‐43^LCD^ was detected using an excitation/emission wavelength of 561/571 nm, while Cy5‐labeled chaperones were detected using an excitation/emission wavelength of 647/657 nm. Data were collected using the fluorescence correlation spectroscopy wizard for a period of 10 s for each acquisition, with 25 technical replicates obtained for each experimental replicate (*n* = 3). Sulforhodamine‐B (SRB) was used for dye calibration due to its known diffusion coefficient of 420 μm^2^/s. Purified GFP was used as a non‐chaperone control and was detected using an excitation/emission wavelength of 488/498 nm.

### 4.15 TCCD data analysis

TCCD data was analyzed using custom written Python scripts (deposited at Zenodo: DOI: 10.5281/zenodo.138587777). Briefly, the background noise within the fluorescence channels for each treatment was determined by fitting the photo count rate (PCF) to a normal distribution. From this, the peak center (P_center_) and full width at half‐maximum (FWHM) were calculated. For each technical replicate, a peak finding algorithm was applied and only those peaks with a PCF higher than the noise threshold (indicative of large oligomeric species) were identified. To ensure that slowly diffusing oligomers were identified as single peaks, the minimum time between peaks was set to 40 ms.

$$\text{Noise threshold}=P_{\text{center}}+\left(2\times \text{FWHM}\right)$$

The number of peaks per replicate and their maximum PCFs (a relative indicator of oligomer size) were then determined. For treatments in which two fluorescence channels were measured simultaneously (e.g., TDP‐43^LCD^ + GFP), the noise fitting and peak finding was performed on each fluorescence channel separately as described above. To determine whether labeled proteins were forming hetero‐oligomers, peaks that were identified in both fluorescence channels within 10 ms of each other were labeled as coincident. Finally, the proportion of TDP‐43^LCD^ peaks that were coincident with sHsp or the non‐chaperone control (i.e., GFP) was determined:

$$\%\mathrm{TDP}-43^{\mathrm{LCD}}\text{coincident}=\frac{\#\;\text{coincident peaks}}{\#\;\mathrm{TDP}-43^{\mathrm{LCD}}\;\text{peaks}}\times 100$$

### 4.16 Statistical analysis

Statistical analysis was performed using Prism software version 10 (GraphPad). Prior to analysis, all data were tested for normal distribution by the Shapiro–Wilk test. Data sets with normal distribution were analyzed by one‐way or two‐way ANOVA. Data sets with non‐normal distribution were analyzed by the Kruskal‐Wallis test.

## AUTHOR CONTRIBUTIONS

**Luke McAlary:** Conceptualization; funding acquisition; writing – review and editing; project administration; resources; supervision. **Thomas B. Walker:** Investigation; writing – original draft; methodology; validation; visualization; writing – review and editing; formal analysis; data curation. **Heath Ecroyd:** Conceptualization; funding acquisition; project administration; resources; supervision; writing – review and editing. **Justin J. Yerbury:** Funding acquisition; conceptualization; supervision; resources; project administration. **Lauren Rice:** Writing – review and editing; formal analysis; investigation; visualization; methodology; validation. **Nicholas Marzano:** Formal analysis; visualization; software; data curation; writing – review and editing. **Shannon McMahon:** Methodology; validation; writing – review and editing. **Joshua W. Trowbridge:** Investigation; methodology; visualization; writing – review and editing; data curation; formal analysis.

## CONFLICT OF INTEREST STATEMENT

The authors declare no conflict of interest.

## Supporting information


**Table S1.** List of bacterial expression vectors. Plasmids are separated by plasmid backbone and further separated by variant. Sources of plasmids are listed with corresponding references.
**Table S2.** Hazard ratios for TDP‐43^LCD^ aggregation in the presence of sHsps. Hazard ratios were calculated from Kaplan–Meier curves in Figures 2, 3, 4 via the log‐rank (Mantel‐Cox) method to determine how each sHsp altered the likelihood of TDP‐43^LCD^ aggregation.
**Figure S1.** High concentrations of TDP‐43^LCD^ exhibit turbidity indicative of condensation.
**Figure S2.** Two‐color coincidence detection (TCCD) was performed using a Leica TCS SP8 FALCON laser scanning confocal microscope. (a‐d) Fluorescence intensity traces of Cy5‐TDP‐43^LCD^ with Alexa Fluor 647‐labeled (a) HspB1^3D^, (b) HspB1^ACD^, (c) HspB5^WT^, and (d) HspB5^ACD^. TDP‐43^LCD^ is indicated in light gray. sHsps are shown in dark gray. Coincident peaks are indicated by orange circles while non‐coincident peaks are indicated by blue circles.
**Figure S3.** HspB1 and HspB5 specifically interact with TDP‐43^LCD^ oligomers Two‐color coincidence detection (TCCD) was performed using a Leica TCS SP8 FALCON laser scanning confocal microscope. (a) Percentage coincidence of chaperones with TDP‐43^LCD^. (b) Percentage coincidence of TDP‐43^LCD^ with chaperones. (c) TDP‐43^LCD^ peak height in the absence or presence of chaperones. (d) Number of TDP‐43^LCD^ peaks in the absence or presence of chaperones. (e) Chaperone peak height in the absence or presence of TDP‐43^LCD^. (f) Number of chaperone peaks in the absence or presence of TDP‐43^LCD^. Data analyzed by Kruskal‐Wallis test with Dunn's post‐hoc test (a, c, d) or two‐way ANOVA with Šidák's post‐hoc test (e, f) (*n* = 75 technical replicates from 3 independent experiments) (* = *p* < 0.05, ** = *p* < 0.01, *** = *p* < 0.001, **** = *p* < 0.0001). No statistical analysis was performed for (b) due to uneven labeling efficiencies.
**Figure S4.** TDP‐43^LCD^ (20 μM) was incubated with mCherry (20 μM) and 200 mM NaCl and imaged using a Leica TCS SP5 laser scanning confocal microscope. Partitioning of mCherry into TDP‐43^LCD^ condensates was not observed. (a) Confocal microscopy images of mCherry partitioning. Scale bar represents 20 μm.

## Data Availability

The data that support the findings of this study are available from the corresponding author upon reasonable request.

## References

[pro70539-bib-0001] Abdolvahabi A , Shi Y , Rasouli S , Croom CM , Aliyan A , Martí AA , et al. Kaplan–Meier meets chemical kinetics: intrinsic rate of SOD1 amyloidogenesis decreased by subset of ALS mutations and cannot fully explain age of disease onset. ACS Chem Nerosci. 2017;8(6):1378–1389. 10.1021/acschemneuro.7b00029 28290665

[pro70539-bib-0002] Ahmad MF , Singh D , Taiyab A , Ramakrishna T , Raman B , Rao CM . Selective Cu2+ binding, redox silencing, and cytoprotective effects of the small heat shock proteins αA‐and αB‐crystallin. J Mol Biol. 2008;382(3):812–824. 18692065 10.1016/j.jmb.2008.07.068

[pro70539-bib-0003] Aquilina JA , Benesch JL , Ding LL , Yaron O , Horwitz J , Robinson CV . Phosphorylation of αB‐crystallin alters chaperone function through loss of dimeric substructure. J Biol Chem. 2004;279(27):28675–28680. 15117944 10.1074/jbc.M403348200

[pro70539-bib-0004] Aquilina JA , Shrestha S , Morris AM , Ecroyd H . Structural and functional aspects of hetero‐oligomers formed by the small heat shock proteins αB‐crystallin and HSP27. J Biol Chem. 2013;288(19):13602–13609. 23532854 10.1074/jbc.M112.443812 PMC3650395

[pro70539-bib-0005] Arai T , Hasegawa M , Akiyama H , Ikeda K , Nonaka T , Mori H , et al. TDP‐43 is a component of ubiquitin‐positive tau‐negative inclusions in frontotemporal lobar degeneration and amyotrophic lateral sclerosis. Biochem Biophys Res Commun. 2006;351(3):602–611. 17084815 10.1016/j.bbrc.2006.10.093

[pro70539-bib-0006] Arrigo AP , Gibert B . Protein interactomes of three stress inducible small heat shock proteins: HspB1, HspB5 and HspB8. Int J Hyperthermia. 2013;29(5):409–422. 23697380 10.3109/02656736.2013.792956

[pro70539-bib-0007] Arseni D , Hasegawa M , Murzin AG , Kametani F , Arai M , Yoshida M , et al. Structure of pathological TDP‐43 filaments from ALS with FTLD. Nature. 2022;601(7891):139–143. 34880495 10.1038/s41586-021-04199-3 PMC7612255

[pro70539-bib-0008] Axelrod D , Koppel DE , Schlessinger J , Elson E , Webb WW . Mobility measurement by analysis of fluorescence photobleaching recovery kinetics. Biophys J. 1976;16(9):1055–1069. 786399 10.1016/S0006-3495(76)85755-4 PMC1334945

[pro70539-bib-0009] Babinchak WM , Haider R , Dumm BK , Sarkar P , Surewicz K , Choi JK , et al. The role of liquid–liquid phase separation in aggregation of the TDP‐43 low‐complexity domain. J Biol Chem. 2019;294(16):6306–6317. 30814253 10.1074/jbc.RA118.007222 PMC6484124

[pro70539-bib-0010] Basha E , O'Neill H , Vierling E . Small heat shock proteins and α‐crystallins: dynamic proteins with flexible functions. Trends Biochem Sci. 2012;37(3):106–117. 22177323 10.1016/j.tibs.2011.11.005 PMC3460807

[pro70539-bib-0011] Benesch JL , Ayoub M , Robinson CV , Aquilina JA . Small heat shock protein activity is regulated by variable oligomeric substructure. J Biol Chem. 2008;283(42):28513–28517. 18713743 10.1074/jbc.M804729200 PMC2661405

[pro70539-bib-0012] Boelens WC . Structural aspects of the human small heat shock proteins related to their functional activities. Cell Stress Chaperones. 2020;25(4):581–591. 32253739 10.1007/s12192-020-01093-1 PMC7332592

[pro70539-bib-0013] Carra S , Alberti S , Arrigo PA , Benesch JL , Benjamin IJ , Boelens W , et al. The growing world of small heat shock proteins: from structure to functions. Cell Stress Chaperones. 2017;22(4):601–611. 28364346 10.1007/s12192-017-0787-8 PMC5465036

[pro70539-bib-0014] Carver JA , Aquilina JA , Truscott RJ , Ralston GB . Identification by 1H NMR spectroscopy of flexible C‐terminal extensions in bovine lens α‐crystallin. FEBS Lett. 1992;311(2):143–149. 1397302 10.1016/0014-5793(92)81386-z

[pro70539-bib-0015] Chappard A , Leighton C , Saleeb RS , Jeacock K , Ball SR , Morris K , et al. Single‐molecule two‐color coincidence detection of unlabeled alpha‐synuclein aggregates. Angew Chem. 2023;135(15):e202216771. 10.1002/ange.202216771 PMC10952349 38516037

[pro70539-bib-0016] Conicella AE , Dignon GL , Zerze GH , Schmidt HB , D'Ordine AM , Kim YC , et al. TDP‐43 α‐helical structure tunes liquid–liquid phase separation and function. Proc Natl Acad Sci USA. 2020;117(11):5883–5894. 32132204 10.1073/pnas.1912055117 PMC7084079

[pro70539-bib-0017] Conicella AE , Zerze GH , Mittal J , Fawzi NL . ALS mutations disrupt phase separation mediated by α‐helical structure in the TDP‐43 low‐complexity C‐terminal domain. Structure. 2016;24(9):1537–1549. 27545621 10.1016/j.str.2016.07.007 PMC5014597

[pro70539-bib-0018] Cox D , Ecroyd H . The small heat shock proteins αB‐crystallin (HSPB5) and Hsp27 (HSPB1) inhibit the intracellular aggregation of α‐synuclein. Cell Stress Chaperones. 2017;22(4):589–600. 28337642 10.1007/s12192-017-0785-x PMC5465035

[pro70539-bib-0019] Cox D , Selig E , Griffin MD , Carver JA , Ecroyd H . Small heat‐shock proteins prevent α‐synuclein aggregation via transient interactions and their efficacy is affected by the rate of aggregation. J Biol Chem. 2016;291(43):22618–22629. 27587396 10.1074/jbc.M116.739250 PMC5077198

[pro70539-bib-0020] Den Engelsman J , Boros S , Dankers PY , Kamps B , Egberts WTV , Böde CS , et al. The small heat‐shock proteins HSPB2 and HSPB3 form well‐defined heterooligomers in a unique 3 to 1 subunit ratio. J Mol Biol. 2009;393(5):1022–1032. 19715703 10.1016/j.jmb.2009.08.052

[pro70539-bib-0021] Ecroyd H , Meehan S , Horwitz J , Aquilina JA , Benesch JL , Robinson CV , et al. Mimicking phosphorylation of αB‐crystallin affects its chaperone activity. Biochem J. 2007;401(1):129–141. 16928191 10.1042/BJ20060981 PMC1698675

[pro70539-bib-0022] Freilich R , Betegon M , Tse E , Mok SA , Julien O , Agard DA , et al. Competing protein‐protein interactions regulate binding of Hsp27 to its client protein tau. Nat Commun. 2018;9(1):4563. 30385828 10.1038/s41467-018-07012-4 PMC6212398

[pro70539-bib-0023] Gasteiger E , Hoogland C , Gattiker A , Duvaud SE , Wilkins MR , Appel RD , et al. Protein identification and analysis tools on the ExPASy server. The proteomics protocols handbook. Totowa, NJ: Humana Press; 2005. p. 571–607.

[pro70539-bib-0024] Guise AJ , Misal SA , Carson R , Chu JH , Boekweg H , Van Der Watt D , et al. TDP‐43‐stratified single‐cell proteomics of postmortem human spinal motor neurons reveals protein dynamics in amyotrophic lateral sclerosis. Cell Rep. 2024;43(1):113636. 38183652 10.1016/j.celrep.2023.113636 PMC10926001

[pro70539-bib-0025] Haider R , Shipley B , Surewicz K , Hinczewski M , Surewicz WK . Pathological C‐terminal phosphomimetic substitutions alter the mechanism of liquid–liquid phase separation of TDP‐43 low complexity domain. Protein Sci. 2024;33(10):e5179. 39302099 10.1002/pro.5179 PMC11413918

[pro70539-bib-0026] Hayes D , Napoli V , Mazurkie A , Stafford WF , Graceffa P . Phosphorylation dependence of hsp27 multimeric size and molecular chaperone function. J Biol Chem. 2009;284(28):18801–18807. 19411251 10.1074/jbc.M109.011353 PMC2707219

[pro70539-bib-0027] Hochberg GK , Ecroyd H , Liu C , Cox D , Cascio D , Sawaya MR , et al. The structured core domain of αB‐crystallin can prevent amyloid fibrillation and associated toxicity. Proc Natl Acad Sci USA. 2014;111(16):E1562–E1570. 24711386 10.1073/pnas.1322673111 PMC4000792

[pro70539-bib-0028] Hubatsch L , Jawerth LM , Love C , Bauermann J , Tang TD , Bo S , et al. Quantitative theory for the diffusive dynamics of liquid condensates. Elife. 2021;10:e68620. 34636323 10.7554/eLife.68620 PMC8580480

[pro70539-bib-0029] Ito H , Kamei K , Iwamoto I , Inaguma Y , Kato K . Regulation of the levels of small heat‐shock proteins during differentiation of C2C12 cells. Exp Cell Res. 2001;266(2):213–221. 11399049 10.1006/excr.2001.5220

[pro70539-bib-0030] Janowska MK , Baughman HE , Woods CN , Klevit RE . Mechanisms of small heat shock proteins. Cold Spring Harb Perspect Biol. 2019;11(10):a034025. 30833458 10.1101/cshperspect.a034025 PMC6771367

[pro70539-bib-0031] Jiang LL , Che MX , Zhao J , Zhou CJ , Xie MY , Li HY , et al. Structural transformation of the amyloidogenic core region of TDP‐43 protein initiates its aggregation and cytoplasmic inclusion. J Biol Chem. 2013;288(27):19614–19624. 23689371 10.1074/jbc.M113.463828 PMC3707662

[pro70539-bib-0032] Johnson BS , Snead D , Lee JJ , McCaffery JM , Shorter J , Gitler AD . TDP‐43 is intrinsically aggregation‐prone, and amyotrophic lateral sclerosis‐linked mutations accelerate aggregation and increase toxicity. J Biol Chem. 2009;284(30):20329–20339. 19465477 10.1074/jbc.M109.010264 PMC2740458

[pro70539-bib-0033] Joosten J , van Sluijs B , Egberts WV , Emmaneel M , Jansen PW , Vermeulen M , et al. Dynamics and composition of small heat shock protein condensates and aggregates. J Mol Biol. 2023;435(13):168139. 37146746 10.1016/j.jmb.2023.168139

[pro70539-bib-0034] Jovcevski B , Kelly MA , Rote AP , Berg T , Gastall HY , Benesch JL , et al. Phosphomimics destabilize Hsp27 oligomeric assemblies and enhance chaperone activity. Chem Biol. 2015;22(2):186–195. 25699602 10.1016/j.chembiol.2015.01.001

[pro70539-bib-0035] Kampinga HH , Hageman J , Vos MJ , Kubota H , Tanguay RM , Bruford EA , et al. Guidelines for the nomenclature of the human heat shock proteins. Cell Stress Chaperones. 2009;14(1):105–111. 18663603 10.1007/s12192-008-0068-7 PMC2673902

[pro70539-bib-0036] Kato S , Hayashi H , Nakashima K , Nanba E , Kato M , Hirano A , et al. Pathological characterization of astrocytic hyaline inclusions in familial amyotrophic lateral sclerosis. Am J Pathol. 1997;151(2):611–620. 9273821 PMC1857998

[pro70539-bib-0037] Kim KK , Kim R , Kim SH . Crystal structure of a small heat‐shock protein. Nature. 1998;394(6693):595–599. 9707123 10.1038/29106

[pro70539-bib-0038] Koulouras G , Panagopoulos A , Rapsomaniki MA , Giakoumakis NN , Taraviras S , Lygerou Z . EasyFRAP‐web: a web‐based tool for the analysis of fluorescence recovery after photobleaching data. Nucleic Acids Res. 2018;46(W1):W467–W472. 29901776 10.1093/nar/gky508 PMC6030846

[pro70539-bib-0039] Laganowsky A , Benesch JL , Landau M , Ding L , Sawaya MR , Cascio D , et al. Crystal structures of truncated alphaA and alphaB crystallins reveal structural mechanisms of polydispersity important for eye lens function. Protein Sci. 2010;19(5):1031–1043. 20440841 10.1002/pro.380 PMC2868245

[pro70539-bib-0040] Li HR , Chen TC , Hsiao CL , Shi L , Chou CY , Huang JR . The physical forces mediating self‐association and phase‐separation in the C‐terminal domain of TDP‐43. Biochim Biophys Acta Proteins Proteom. 2018;1866(2):214–223. 28988034 10.1016/j.bbapap.2017.10.001

[pro70539-bib-0041] Li HR , Chiang WC , Chou PC , Wang WJ , Huang JR . TAR DNA‐binding protein 43 (TDP‐43) liquid–liquid phase separation is mediated by just a few aromatic residues. J Biol Chem. 2018;293(16):6090–6098. 29511089 10.1074/jbc.AC117.001037 PMC5912450

[pro70539-bib-0042] Ling SC , Polymenidou M , Cleveland DW . Converging mechanisms in ALS and FTD: disrupted RNA and protein homeostasis. Neuron. 2013;79(3):416–438. 23931993 10.1016/j.neuron.2013.07.033 PMC4411085

[pro70539-bib-0043] Lu S , Hu J , Arogundade OA , Goginashvili A , Vazquez‐Sanchez S , Diedrich JK , et al. Heat‐shock chaperone HSPB1 regulates cytoplasmic TDP‐43 phase separation and liquid‐to‐gel transition. Nat Cell Biol. 2022;24(9):1378–1393. 36075972 10.1038/s41556-022-00988-8 PMC9872726

[pro70539-bib-0044] Mainz A , Peschek J , Stavropoulou M , Back KC , Bardiaux B , Asami S , et al. The chaperone αB‐crystallin uses different interfaces to capture an amorphous and an amyloid client. Nat Struct Mol Biol. 2015;22(11):898–905. 26458046 10.1038/nsmb.3108

[pro70539-bib-0045] Mann JR , Gleixner AM , Mauna JC , Gomes E , DeChellis‐Marks MR , Needham PG , et al. RNA binding antagonizes neurotoxic phase transitions of TDP‐43. Neuron. 2019;102(2):321–338. 30826182 10.1016/j.neuron.2019.01.048 PMC6472983

[pro70539-bib-0046] McAlary L , Plotkin SS , Yerbury JJ , Cashman NR . Prion‐like propagation of protein misfolding and aggregation in amyotrophic lateral sclerosis. Front Mol Neurosci. 2019;12:262. 31736708 10.3389/fnmol.2019.00262 PMC6838634

[pro70539-bib-0047] McCall PM , Kim K , Shevchenko A , Ruer‐Gruß M , Peychl J , Guck J , et al. A label‐free method for measuring the composition of multicomponent biomolecular condensates. Nat Chem. 2025;17(12):1. 40903498 10.1038/s41557-025-01928-3 PMC12669041

[pro70539-bib-0048] McDonald ET , Bortolus M , Koteiche HA , Mchaourab HS . Sequence, structure, and dynamic determinants of Hsp27 (HspB1) equilibrium dissociation are encoded by the N‐terminal domain. Biochemistry. 2012;51(6):1257–1268. 22264079 10.1021/bi2017624 PMC3293247

[pro70539-bib-0049] Mohanty P , Shenoy J , Rizuan A , Jovic N , Mercado‐Ortiz J , Fawzi NL , et al. Hydrophobic residues in disordered and helical regions mediate the oligomerization and phase separation of TDP‐43 C‐terminal domain. Biophys J. 2023;122(3):208a.

[pro70539-bib-0050] Mohanty P , Shenoy J , Rizuan A , Mercado‐Ortiz JF , Fawzi NL , Mittal J . A synergy between site‐specific and transient interactions drives the phase separation of a disordered, low‐complexity domain. Proc Natl Acad Sci USA. 2023;120(34):e2305625120. 37579155 10.1073/pnas.2305625120 PMC10450430

[pro70539-bib-0051] Morelli FF , Verbeek DS , Bertacchini J , Vinet J , Mediani L , Marmiroli S , et al. Aberrant compartment formation by HSPB2 mislocalizes lamin A and compromises nuclear integrity and function. Cell Rep. 2017;20(9):2100–2115. 28854361 10.1016/j.celrep.2017.08.018 PMC5583511

[pro70539-bib-0052] Mymrikov EV , Riedl M , Peters C , Weinkauf S , Haslbeck M , Buchner J . Regulation of small heat‐shock proteins by hetero‐oligomer formation. J Biol Chem. 2020;295(1):158–169. 31767683 10.1074/jbc.RA119.011143 PMC6952609

[pro70539-bib-0053] Nakamoto H , Vigh L . The small heat shock proteins and their clients. Cell Mol Life Sci. 2007;64:294–306. 17187175 10.1007/s00018-006-6321-2 PMC11138444

[pro70539-bib-0054] Nielsen L , Khurana R , Coats A , Frokjaer S , Brange J , Vyas S , et al. Effect of environmental factors on the kinetics of insulin fibril formation: elucidation of the molecular mechanism. Biochemistry. 2001;40(20):6036–6046. 11352739 10.1021/bi002555c

[pro70539-bib-0055] Pakravan D , Michiels E , Bratek‐Skicki A , De Decker M , Van Lindt J , Alsteens D , et al. Liquid–liquid phase separation enhances TDP‐43 LCD aggregation but delays seeded aggregation. Biomolecules. 2021;11(4):548. 33917983 10.3390/biom11040548 PMC8068378

[pro70539-bib-0056] Pantoja‐Uceda D , Stuani C , Laurents DV , McDermott AE , Buratti E , Mompeán M . Phe‐Gly motifs drive fibrillization of TDP‐43's prion‐like domain condensates. PLoS Biol. 2021;19(4):e3001198. 33909608 10.1371/journal.pbio.3001198 PMC8109789

[pro70539-bib-0057] Peschek J , Braun N , Rohrberg J , Back KC , Kriehuber T , Kastenmüller A , et al. Regulated structural transitions unleash the chaperone activity of αB‐crystallin. Proc Natl Acad Sci USA. 2013;110(40):E3780–E3789. 24043785 10.1073/pnas.1308898110 PMC3791731

[pro70539-bib-0058] Prasad A , Bharathi V , Sivalingam V , Girdhar A , Patel BK . Molecular mechanisms of TDP‐43 misfolding and pathology in amyotrophic lateral sclerosis. Front Mol Neurosci. 2019;12:25. 30837838 10.3389/fnmol.2019.00025 PMC6382748

[pro70539-bib-0059] Rambaran RN , Serpell LC . Amyloid fibrils: abnormal protein assembly. Prion. 2008;2(3):112–117. 19158505 10.4161/pri.2.3.7488 PMC2634529

[pro70539-bib-0060] Selig EE , Zlatic CO , Cox D , Mok YF , Gooley PR , Ecroyd H , et al. N‐and C‐terminal regions of αB‐crystallin and Hsp27 mediate inhibition of amyloid nucleation, fibril binding, and fibril disaggregation. J Biol Chem. 2020;295(29):9838–9854. 32417755 10.1074/jbc.RA120.012748 PMC7380184

[pro70539-bib-0061] Sharp PS , Akbar MT , Bouri S , Senda A , Joshi K , Chen HJ , et al. Protective effects of heat shock protein 27 in a model of ALS occur in the early stages of disease progression. Neurobiol Dis. 2008;30(1):42–55. 18255302 10.1016/j.nbd.2007.12.002

[pro70539-bib-0062] Staderini T , Bigi A , Lagrève C , Marzi I , Bemporad F , Chiti F . Biophysical characterization of the phase separation of TDP‐43 devoid of the C‐terminal domain. Cell Mol Biol Lett. 2024;29(1):104. 38997630 10.1186/s11658-024-00615-4 PMC11245819

[pro70539-bib-0063] Staderini T , Bigi A , Mongiello D , Cecchi C , Chiti F . Biophysical characterization of full‐length TAR DNA‐binding protein (TDP‐43) phase separation. Protein Sci. 2022;31(12):e4509. 36371546 10.1002/pro.4509 PMC9703588

[pro70539-bib-0064] Tamaki Y , Urushitani M . Molecular dissection of TDP‐43 as a leading cause of ALS/FTLD. Int J Mol Sci. 2022;23(20):12508. 36293362 10.3390/ijms232012508 PMC9604209

[pro70539-bib-0065] Tedesco B , Cristofani R , Ferrari V , Cozzi M , Rusmini P , Casarotto E , et al. Insights on human small heat shock proteins and their alterations in diseases. Front Mol Biosci. 2022;9:842149. 35281256 10.3389/fmolb.2022.842149 PMC8913478

[pro70539-bib-0066] Van Montfort RL , Basha E , Friedrich KL , Slingsby C , Vierling E . Crystal structure and assembly of a eukaryotic small heat shock protein. Nat Struct Biol. 2001;8(12):1025–1030. 11702068 10.1038/nsb722

[pro70539-bib-0067] Vendruscolo M , Fuxreiter M . Protein condensation diseases: therapeutic opportunities. Nat Commun. 2022;13(1):5550. 36138006 10.1038/s41467-022-32940-7 PMC9500012

[pro70539-bib-0068] Yamashita T , Yokota O , Ousaka D , Sun H , Haraguchi T , Ota‐Elliott RS , et al. Biallelic variants in DNAJC7 cause familial amyotrophic lateral sclerosis with the TDP‐43 pathology. Acta Neuropathol. 2025;150(1):1–24. 40802071 10.1007/s00401-025-02899-y PMC12350594

[pro70539-bib-0069] Yerbury JJ , Farrawell NE , McAlary L . Proteome homeostasis dysfunction: a unifying principle in ALS pathogenesis. Trends Neurosci. 2020;43(5):274–284. 32353332 10.1016/j.tins.2020.03.002

