# Supplementary material for: Small heat shock proteins HspB1 and HspB5 differentially alter the condensation and aggregation of the TDP‐43 low‐complexity domain
Source: Protein Sci. 2026 Mar 19;35(4):e70539. doi: 10.1002/pro.70539 (PMC13140710; doi:10.1002/pro.70539)
Supplement: Supplementary file 1 — Table S1. List of bacterial expression vectors. Plasmids are separated by plasmid backbone and further separated by variant. Sources of plasmids are listed with corresponding references. Table S2. Hazard ratios for TDP‐43LCD aggregation in the presence of sHsps. Hazard ratios were calculated from Kaplan–Meier curves in Figures 2, 3, 4 via the log‐rank (Mantel‐Cox) method to determine how each sHsp altered the likelihood of TDP‐43LCD aggregation. Figure S1. High concentrations of TDP‐43LCD exhibit turbidity indicative of condensation. Figure S2. Two‐color coincidence detection (TCCD) was performed using a Leica TCS SP8 FALCON laser scanning confocal microscope. (a‐d) Fluorescence intensity traces of Cy5‐TDP‐43LCD with Alexa Fluor 647‐labeled (a) HspB13D, (b) HspB1ACD, (c) HspB5WT, and (d) HspB5ACD. TDP‐43LCD is indicated in light gray. sHsps are shown in dark gray. Coincident peaks are indicated by orange circles while non‐coincident peaks are indicated by blue circles. Figure S3. HspB1 and HspB5 specifically interact with TDP‐43LCD oligomers Two‐color coincidence detection (TCCD) was performed using a Leica TCS SP8 FALCON laser scanning confocal microscope. (a) Percentage coincidence of chaperones with TDP‐43LCD. (b) Percentage coincidence of TDP‐43LCD with chaperones. (c) TDP‐43LCD peak height in the absence or presence of chaperones. (d) Number of TDP‐43LCD peaks in the absence or presence of chaperones. (e) Chaperone peak height in the absence or presence of TDP‐43LCD. (f) Number of chaperone peaks in the absence or presence of TDP‐43LCD. Data analyzed by Kruskal‐Wallis test with Dunn's post‐hoc test (a, c, d) or two‐way ANOVA with Šidák's post‐hoc test (e, f) (n = 75 technical replicates from 3 independent experiments) (* = p < 0.05, ** = p < 0.01, *** = p < 0.001, **** = p < 0.0001). No statistical analysis was performed for (b) due to uneven labeling efficiencies. Figure S4. TDP‐43LCD (20 μM) was incubated with mCherry (20 μM) and 200 mM NaCl and imaged using [file PRO-35-e70539-s001.docx]

**Small heat shock proteins HspB1 and HspB5 differentially alter the condensation and aggregation of the TDP-43 low complexity domain**

*Thomas B. Walker^1^, Joshua W. Trowbridge^1^,* *Shannon McMahon^1^, Nicholas Marzano^1^, Lauren Rice^1^, Justin J. Yerbury^1^, Heath Ecroyd^1^, Luke McAlary^1^*

*^1^University of Wollongong, Australia*

*Corresponding author: Luke McAlary, Northfields Avenue, Wollongong, New South Wales, Australia 2522; +61242211406;* [*lmcalary@uow.edu.au*](mailto:lmcalary@uow.edu.au)

**Supplementary material description**

**Supplementary Table 1**

**Supplementary Table 1. List of bacterial expression vectors.** Plasmids are separated by plasmid backbone and further separated by variant. Sources of plasmids are listed with corresponding references.

| ***Bacterial expression vector***  pJ411 TDP-43_CTD Contains the gene for bacterial expression of the TDP-43 low complexity domain (LCD) with a 6His-tag and TEV protease site at its N- terminus. The antibiotic resistance is Kan^r^. | | |
| --- | --- | --- |
| ***Bacterial expression vector***  pET3a HspB1 Contains the gene for bacterial expression of HspB1. The antibiotic resistance is Amp^r^. | | |
| **Variants used in study**  HspB1^WT^  HspB1^3D^ | **Source**  Ecroyd group (Wollongong, AU)  Ecroyd group (Wollongong, AU) | **Reference**  (28)  (28) |
| ***Bacterial expression vector***  pET24a HspB5 Contains the gene for bacterial expression of HspB1. The antibiotic resistance is Kan^r^. | | |
| **Variants used in study**  HspB5^WT^  HspB5^3D^ | **Source**  Ecroyd group (Wollongong, AU)  Ecroyd group (Wollongong, AU) | **Reference**  (21)  (21) |
| ***Bacterial expression vector***  pET28-HSP_ACD Contains the gene for bacterial expression of the α-crystallin domain (ACD) of HspB1 or HspB5. The antibiotic resistance is Kan^r^. | | |
| **Variants used in study**  HspB1^ACD^  HspB5^ACD^ | **Source**  Laganowsky group (Texas A&M Health Science Center, USA)  Laganowsky group (Texas A&M Health Science Center, USA) | **Reference**  (65)  (65) |

**Supplementary Table 2**

**Supplementary Table 1. Hazard ratios for TDP-43^LCD^ aggregation in the presence of sHsps.** Hazard ratios were calculated from Kaplan-Meier curves in Figures 2-4 via the log-rank (Mantel-Cox) method to determine how each sHsp altered the likelihood of TDP-43^LCD^ aggregation.

| Molar ratio | HspB1^WT^ | | HspB1^3D^ | | HspB1^ACD^ | | HspB5^WT^ | | HspB5^3D^ | | HspB5^ACD^ | |
| --- | --- | --- | --- | --- | --- | --- | --- | --- | --- | --- | --- | --- |
|  | Hazard ratio | *P* | Hazard ratio | *P* | Hazard ratio | *P* | Hazard ratio | *P* | Hazard ratio | *P* | Hazard ratio | *P* |
| 5:1 | 0.1985 | <0.0001 | 4.000 | 0.0002 | *N/A* | *N/A* | 0.1026 | <0.0001 | 0.1848 | <0.0001 | *N/A* | *N/A* |
| 1:1 | 0.2057 | <0.0001 | 0.2515 | 0.0002 | 0.4884 | ns | 0.1694 | <0.0001 | 0.1848 | <0.0001 | 0.9334 | ns |
| 1:5 | 0.2124 | <0.0001 | 0.2267 | 0.0002 | *N/A* | *N/A* | 0.1417 | <0.0001 | 0.1848 | <0.0001 | *N/A* | *N/A* |
| 1:10 | 0.2268 | <0.0001 | 0.2610 | 0.00035 | 1.2520 | ns | 0.1417 | <0.0001 | 0.1848 | <0.0001 | 1.4070 | ns |
| 1:100 | 0.5383 | ns | 0.2918 | 0.0423 | 1.2780 | ns | 0.2834 | 0.0005 | 0.2100 | <0.0001 | 3.4980 | 0.0014 |

**Supplementary Figure 1**

TDP-43^LCD^ incubated at 50 μM rapidly became turbid, which was indicative of it having undergone condensation at these concentrations (**Fig. S1a**). The kinetics of TDP-43^LCD^ aggregation at this concentration and at 20 μM were enhanced compared to 10 μM TDP-43^LCD^ (**Fig. 1**), indicating that TDP-43^LCD^ condensation promotes its aggregation.


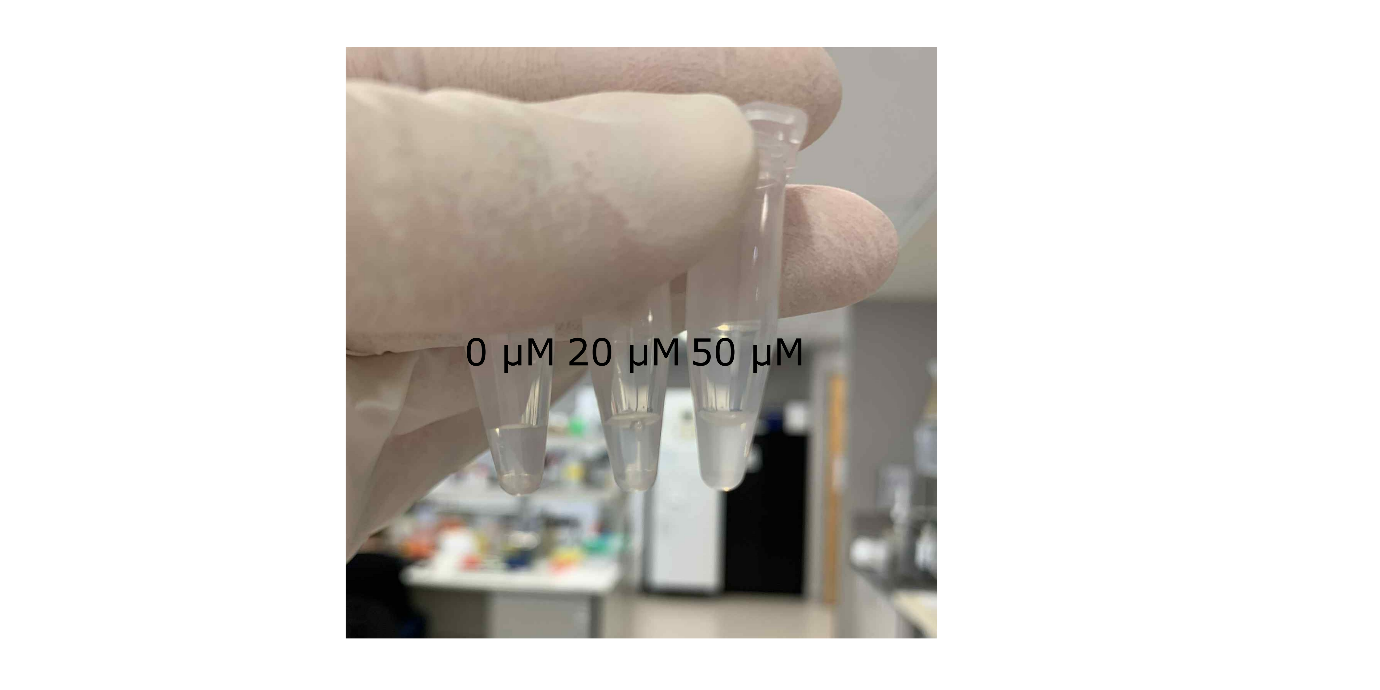


**Supplementary Figure 1.** High concentrations of TDP-43^LCD^ exhibit turbidity indicative of condensation.

**Supplementary Figure 2**


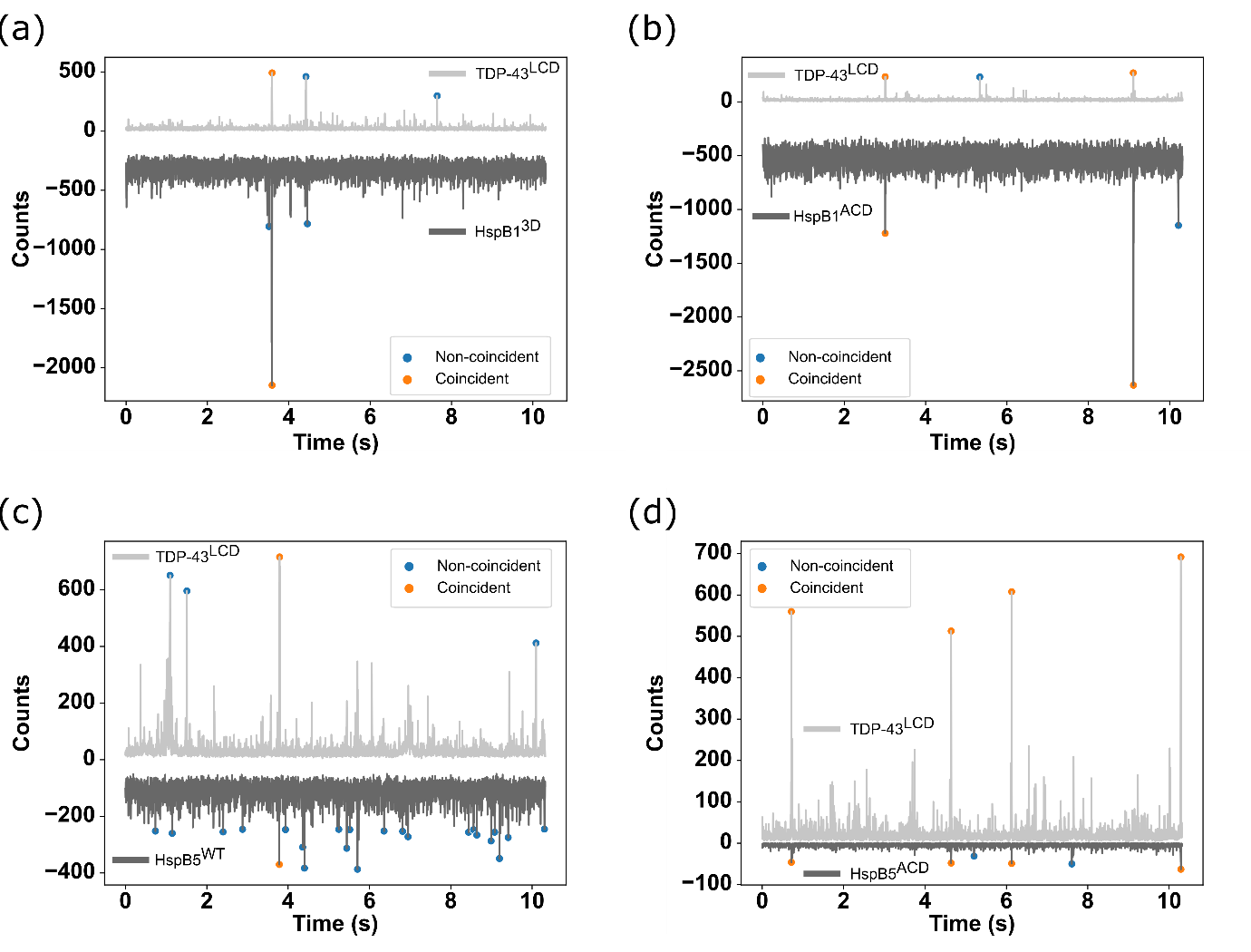


**Supplementary Figure 2.** Two-colour coincidence detection (TCCD) was performed using a Leica TCS SP8 FALCON laser scanning confocal microscope. (a-d) Fluorescence intensity traces of Cy5-TDP-43^LCD^ with Alexa Fluor 647-labelled (a) HspB1^3D^, (b) HspB1^ACD^, (c) HspB5^WT^, and (d) HspB5^ACD^. TDP-43^LCD^ is indicated in light grey. sHsps are shown in dark grey. Coincident peaks are indicated by orange circles while non-coincident peaks are indicated by blue circles.

**Supplementary Figure 3**

Since both HspB1 and HspB5 delayed the formation of ThT-positive TDP-43^LCD^ fibrils, we hypothesised that they do so by interacting with monomers or small oligomers of TDP-43^LCD^. To investigate this, we performed two-colour coincidence detection (TCCD) experiments using Cy5-labelled TDP-43^LCD^ and Alexa Fluor 647-labelled sHsp isoforms (69). TCCD experiments rely upon at least two differently fluorescently-labelled particles diffusing through a confocal volume either together or not. To do this, TDP-43^LCD^ was either incubated alone or in the presence of sHsp variants under identical conditions as described in the assays above. When measuring diffusion of Cy5-labelled TDP-43^LCD^ and Alexa Fluor 647-labelled chaperones through the confocal volume, we frequently observed large peaks in fluorescence intensity that were significantly greater than background intensity (**Fig. S2**). We hypothesised that smaller peaks in Cy5 fluorescence represent monomeric TDP-43^LCD^, while larger peaks in fluorescence intensity represent higher order states such as oligomers and larger aggregates. Notably, peaks in fluorescence intensity corresponding to Alexa Fluor 647-labelled chaperones often were observed at concurrently to peaks in Cy5 fluorescence (**Fig. S2**) Therefore, we sought to quantify the coincidence of fluorescence intensity peaks from Cy3-labelled TDP-43^LCD^ with those observed from Alexa Fluor 647-labelled chaperones, and vice versa.

When TDP-43^LCD^ was incubated with the sHsps, TDP-43^LCD^ peaks were found to be coincident with peaks from the molecular chaperones significantly more often compared to when incubated with the non-chaperone control protein, GFP, indicating that TDP-43^LCD^ interacts specifically with the chaperones (**Fig. S3a**). Conversely, sHsps peaks, but not GFP, were coincident with TDP-43^LCD^ peaks (**Fig. S3a**). Critically, the addition of 6 M guanidine-HCl to these samples resulted in a significant reduction in the size and number of TDP-43^LCD^ peaks, such that they were virtually absent, confirming that these peaks indeed represented aggregated species (**Fig. S3c, d**). TDP-43^LCD^ peak height (which is an indicator of oligomeric size) was reduced in the presence of all sHsp isoforms except HspB5^WT^, suggesting that incubation of TDP-43^LCD^ with these chaperones reduces the size of TDP-43^LCD^ oligomers (**Fig. 5c**). Furthermore, the number of TDP-43^LCD^ peaks was significantly reduced in the presence of each of the chaperones, as well as GFP, suggesting that less TDP-43^LCD^ oligomers are formed upon co-incubation (**Fig. S3d**).

When we looked at the fluorescence intensity originating from the chaperones that were coincident with TDP-43^LCD^, we found that the peak height was reduced only when TDP-43^LCD^ was co-incubated with HspB5^WT^ (**Fig S3e**). We observed a significant increase in the number of HspB1^3D^ and HspB5^WT^ peaks when they were in the presence of TDP-43^LCD^, suggesting that they were being recruited to TDP-43^LCD^ aggregates (**Fig. S3f**). In contrast, there were fewer HspB5^ACD^ peaks in the presence of TDP-43^LCD^ (**Fig. S3f**). Together, these results indicate that HspB1 and HspB5 specifically bind to TDP-43^LCD^ oligomers through interactions that involve their core ACDs and, by doing so, inhibit the assembly of TDP-43^LCD^ oligomers into higher order aggregates.

**
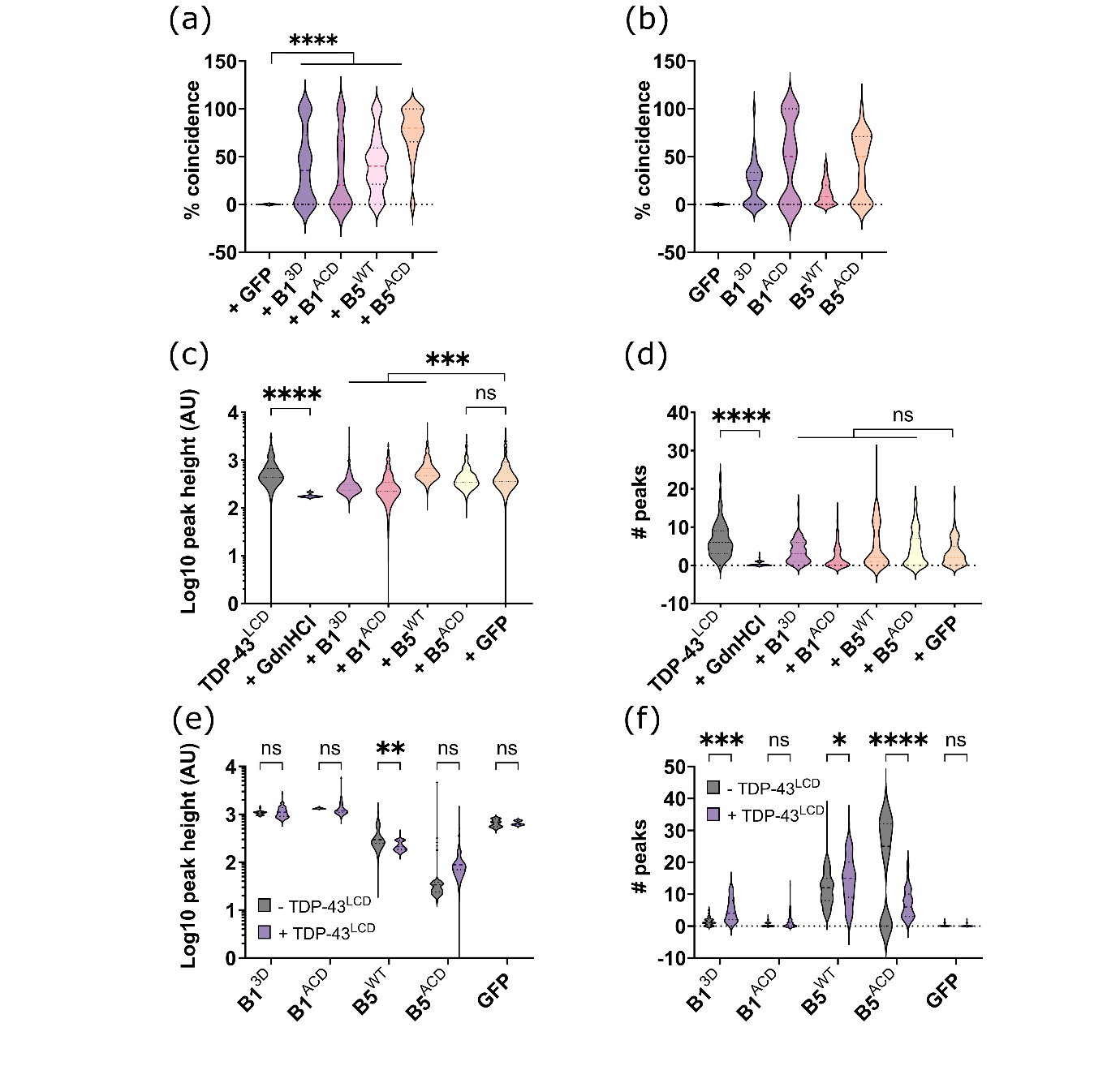
**

**Supplementary Figure 3. HspB1 and HspB5 specifically interact with TDP-43^LCD^ oligomers** Two-colour coincidence detection (TCCD) was performed using a Leica TCS SP8 FALCON laser scanning confocal microscope. (a) Percentage coincidence of chaperones with TDP-43^LCD^. (b) Percentage coincidence of TDP-43^LCD^ with chaperones. (c) TDP-43^LCD^ peak height in the absence or presence of chaperones. (d) Number of TDP-43^LCD^ peaks in the absence or presence of chaperones. (e) Chaperone peak height in the absence or presence of TDP-43^LCD^. (f) Number of chaperone peaks in the absence or presence of TDP-43^LCD^. Data analysed by Kruskal-Wallis test with Dunn’s post-hoc test (a, c, d) or two-way ANOVA with Šidák’s post-hoc test (e, f) (n = 75 technical replicates from 3 independent experiments) (* = P < 0.05, ** = P < 0.01, *** = P < 0.001, **** = P < 0.0001). No statistical analysis was performed for (b) due to uneven labelling efficiencies.

**Supplementary Figure 4**

To determine whether partitioning to TDP-43^LCD^ condensates was a specific property of HspB1 and HspB5, TDP-43^LCD^ was incubated with the non-chaperone protein mCherry and 200 mM NaCl. As expected, mCherry was excluded from TDP-43^LCD^ condensates (**Fig. S4a**).


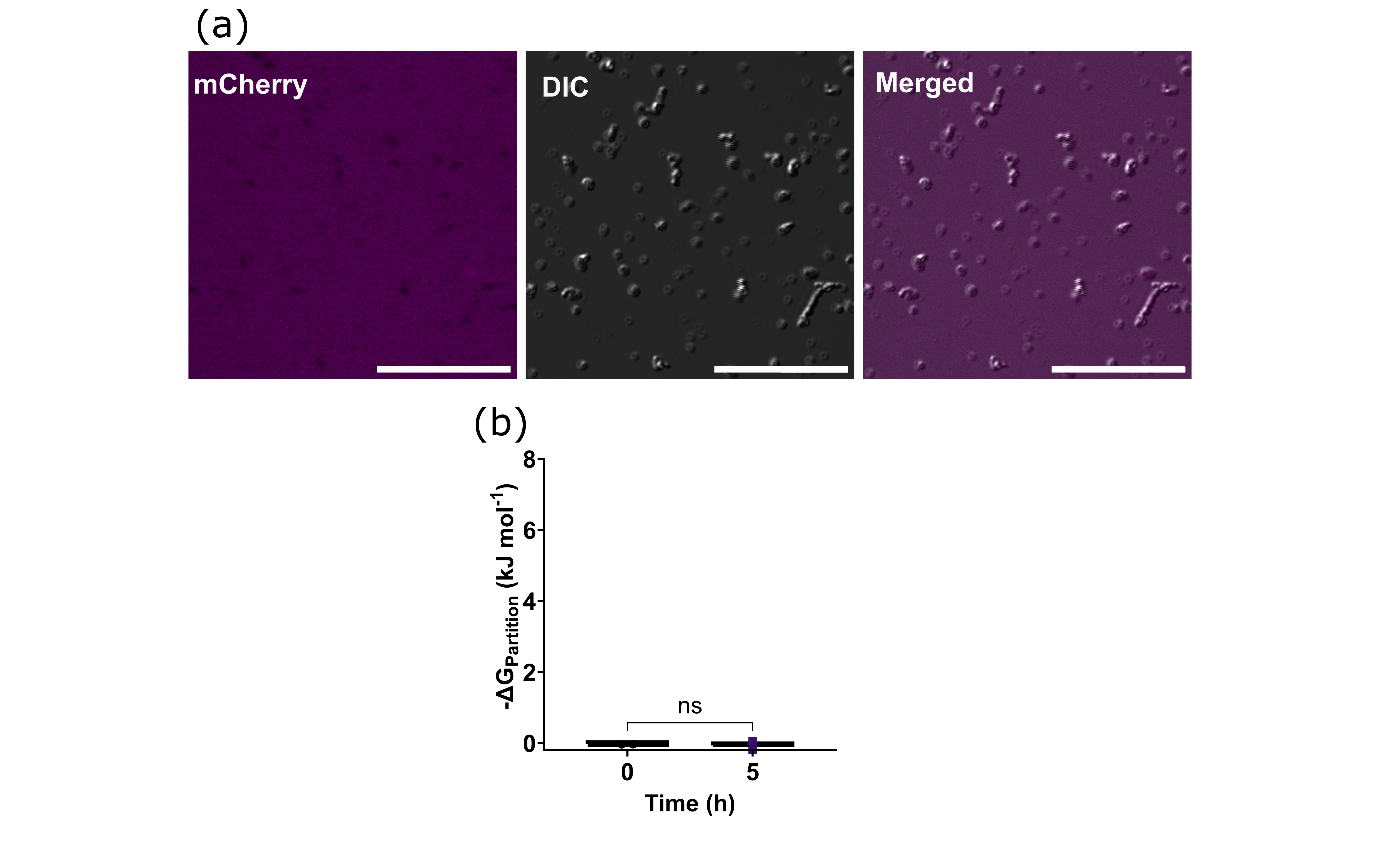


**Supplementary Figure 4.** TDP-43^LCD^ (20 μM) was incubated with mCherry (20 μM) and 200 mM NaCl and imaged using a Leica TCS SP5 laser scanning confocal microscope. Partitioning of mCherry into TDP-43^LCD^ condensates was not observed. (a) Confocal microscopy images of mCherry partitioning. Scale bar represents 20 μm.
